# Unveiling the immunomodulatory shift: Epithelial-mesenchymal transition Alters immune mechanisms of amniotic epithelial cells

**DOI:** 10.1016/j.isci.2023.107582

**Published:** 2023-08-09

**Authors:** Valeria Di Lollo, Angelo Canciello, Alessia Peserico, Massimiliano Orsini, Valentina Russo, Adrián Cerveró-Varona, Beatrice Dufrusine, Mohammad El Khatib, Valentina Curini, Annunziata Mauro, Paolo Berardinelli, Cathy Tournier, Massimo Ancora, Cesare Cammà, Enrico Dainese, Luana Fiorella Mincarelli, Barbara Barboni

**Affiliations:** 1 Department of Biosciences and Technology for Food, Agriculture and Environment, University of Teramo, Via Balzarini 1, 64100 Teramo, Italy; 2 National Reference Center for Whole Genome Sequencing of Microbial Pathogens: Database and Bioinformatic Analysis, Istituto Zooprofilattico Sperimentale dell'Abruzzo e del Molise, Campo Boario, 64100 Teramo, Italy; 3 Istituto Zooprofilattico Sperimentale delle Venezie, Department of Microbiology, Viale dell’Università 10, 35020 Legnaro (PD), Italy; 4 Division of Cancer Sciences, School of Medical Sciences, Faculty of Biology, Medicine and Health, University of Manchester, Manchester M13 9PL, UK

**Keywords:** Immunology, Cell biology, Stem cells research, Transcriptomics

## Abstract

Epithelial-mesenchymal transition (EMT) changes cell phenotype by affecting immune properties of amniotic epithelial cells (AECs). The present study shows how the response to lipopolysaccharide of cells collected pre- (eAECs) and post-EMT (mAECs) induces changes in their transcriptomics profile. In fact, eAECs mainly upregulate genes involved in antigen-presenting response, whereas mAECs over-express soluble inflammatory mediator transcripts. Consistently, network analysis identifies CIITA and Nrf2 as main drivers of eAECs and mAECs immune response, respectively. As a consequence, the depletion of CIITA and Nrf2 impairs the ability of eAECs and mAECs to inhibit lymphocyte proliferation or macrophage-dependent IL-6 release, thus confirming their involvement in regulating immune response. Deciphering the mechanisms controlling the immune function of AECs pre- and post-EMT represents a step forward in understanding key physiological events wherein these cells are involved (pregnancy and labor). Moreover, controlling the immunomodulatory properties of eAECs and mAECs may be essential in developing potential strategies for regenerative medicine applications.

## Highlights

• EMT influences the immune behavior of AECs
• CIITA and Nrf2 control the immune response of eAECs (pre-EMT) and mAECs (post-EMT)
• eAECs harness the antigen-presenting response to suppress inflammation
• mAECs exhibit both anti- and pro-inflammatory functions to mitigate inflammation

## Graphical abstract

**Figure:**
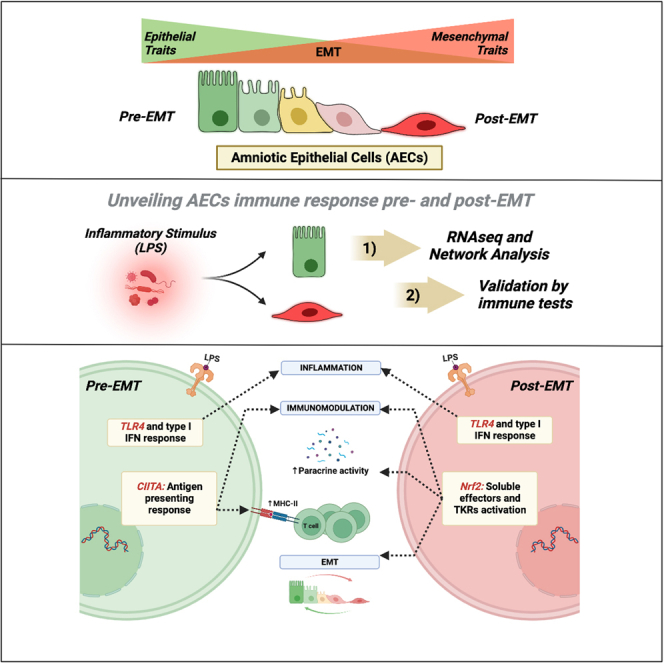

## Introduction

Epithelial-mesenchymal transition (EMT) is a multi-step reversible process during which epithelial cells gradually undergo phenotypic changes to eventually acquire mesenchymal shape and features.^1^^,^^2^ Generally, EMT occurs during early embryonic development, fibrosis, and metastatic transformation. Growing evidence demonstrated how this process, and its counterpart, mesenchymal-epithelial transition, MET, is constantly regulated by multiple physiological and pathological conditions.^3^^,^^4^^,^^5^^,^^6^ EMT also affects cell immune response and plays a pivotal role in tumor immunosuppression and immune evasion processes.^7^^,^^8^^,^^9^^,^^10^

Epithelial stem cells show native immune properties since they provide the first line of defense against microenvironment perturbations in several tissues, such as skin,^11^^,^^12^^,^^13^ lung,^14^ placenta,^15^ and intestine.^16^ In this regard, amniotic membrane (AM) has been historically considered an important medical device with many applications in regenerative medicine for its structural and biological properties.^17^ In particular, the widespread use of AM depends on its anti-inflammatory, anti-bacterial, anti-microbial, anti-fibrotic, and anti-cancer properties and, nevertheless, for its huge immunomodulatory potential. The latter effect is further important in the field of cell-based therapy and mostly depends on the immunological advantages of both cell populations that form AM: amniotic epithelial cells (AECs) and amniotic mesenchymal cells (AMCs).^15^^,^^18^^,^^19^^,^^20^ Intriguingly, we and others demonstrated that the contribution of AECs and AMCs to immunological properties of AM is substantially different.^21^^,^^22^ Accordingly, AECs themselves significantly change their immunomodulatory properties following EMT.^23^^,^^24^^,^^25^ Therefore, an undoubted correlation between AECs phenotype and biological properties is consistent, even though the underlying molecular mechanisms are not yet elucidated. This is an important aspect to be considered because EMT spontaneously affects AECs both *in vivo* and *in vitro*, leading to the generation of two distinct cell populations: (1) AECs retaining native epithelial phenotype and (2) EMT-derived AECs acquiring mesenchymal phenotype (hereafter referred as eAECs and mAECs, respectively). Physiologically, AECs undergo EMT at labor in order to weaken AM in preparation for delivery^26^; on the other hand, AECs could experience EMT as a consequence of *in vitro* culture and the loss of progesterone (or other placental factors). This latter hypothesis is strongly supported by the fact that *in vitro* supplementation this hormone either preserves eAECs epithelial phenotype or induces MET in mAECs.^20^^,^^23^^,^^24^

Based on this premise, understanding whether and how EMT could modulate AECs immune properties would be beneficial to exploit these cells in therapy. To this aim, we performed RNA sequencing (RNA-seq) to identify the key gene regulators of the immune response of AECs, which were subsequently validated though functional immune tests.

## Results

### Transcriptomics landscape of AECs immune response

The native epithelial phenotype of AECs can be preserved by progesterone treatment,^20^ thus preventing EMT and giving rise to two distinct cell populations: eAECs and mAECs. Phenotypic and molecular changes imposed by EMT were reported in Figure S1. To this regard, the addition of progesterone to the culture for three passages preserved approximately 80% positivity for epithelial marker (eAECs), whereas EMT, which spontaneously occurred within AECs after three passages, induced an enrichment of more than 80% of mesenchymal cells (mAECs).^23^

Here, we investigated how amniotic-derived cells (in both epithelial and mesenchymal phenotype) respond to inflammation insults. The transcriptome of *in vitro* expanded AECs in presence of P4 (eAECs) and without P4 (mAECs) was analyzed upon treatment with lipopolysaccharide (LPS) by means of RNA-seq analysis.

Transcriptome sequencing returned from 28.2 to 35.2 million of clean reads with an alignment rate, calculated by Kallisto aligner^27^ against the reference transcriptome (OviAries 3.1 sheep genome, Ensembl 99), ranging from 44.8% to 49.1% reporting a large amount of not annotated transcripts (Table S1). The total collection included 19,738 transcripts for which genes were found.

A total of 360 and 315 differentially expressed genes (DEGs) were recognized, respectively, in LPS-stimulated eAECs and mAECs when compared with their not-stimulated counterpart. Among them, 273 and 232 genes were over-expressed while 87 and 83 were under-expressed, respectively, with most of them involved in cell motility (EMILIN2, TBCD, IFT74, COL18A1, DLGAP5 under-expressed in LPS-eAECs; MYO15B, ELMO3, FERMT1 under-expressed in LPS-mAECs), immune stress response (B2M, IL6, ZCWPW1, DSC2, RPL35A, PSMD14, HMGN over-expressed in LPS-eAECs), and cell signaling processes (PI4KB, GPR151, PLEKHH2, SLC38A10 under-expressed in LPS-eAECs; ISG15, MX1, JAG1 over-expressed in LPS-mAECs) (Table S2).

Furthermore, the comparison of the two LPS-stimulated cell populations showed 270 DEGs (182 over-expressed and 88 under-expressed) (Table S2). In detail, under LPS stimulation, mAECs mainly activated mechanisms of cell defense by the upregulation of genes belonging to the proteasomal degradation machinery (TSPAN3, POMP, CPXM1, UBL4A, BUB1) but downregulated genes related to vesicular protein trafficking (TMED4) and mechanisms of molecular transport across the cell membrane (ATP1A4, WNK4).

Gene ontology (GO) enrichment analysis indicated that eAECs and mAECs respond to LPS stimulus by activating common biological processes including nitrogen metabolic processes, macromolecule metabolism, and translational activities. Interestingly, GO analysis revealed divergent behavior of eAECs and mAECs in response to LPS (Table S3 and Data S1).

**Figure 1:**
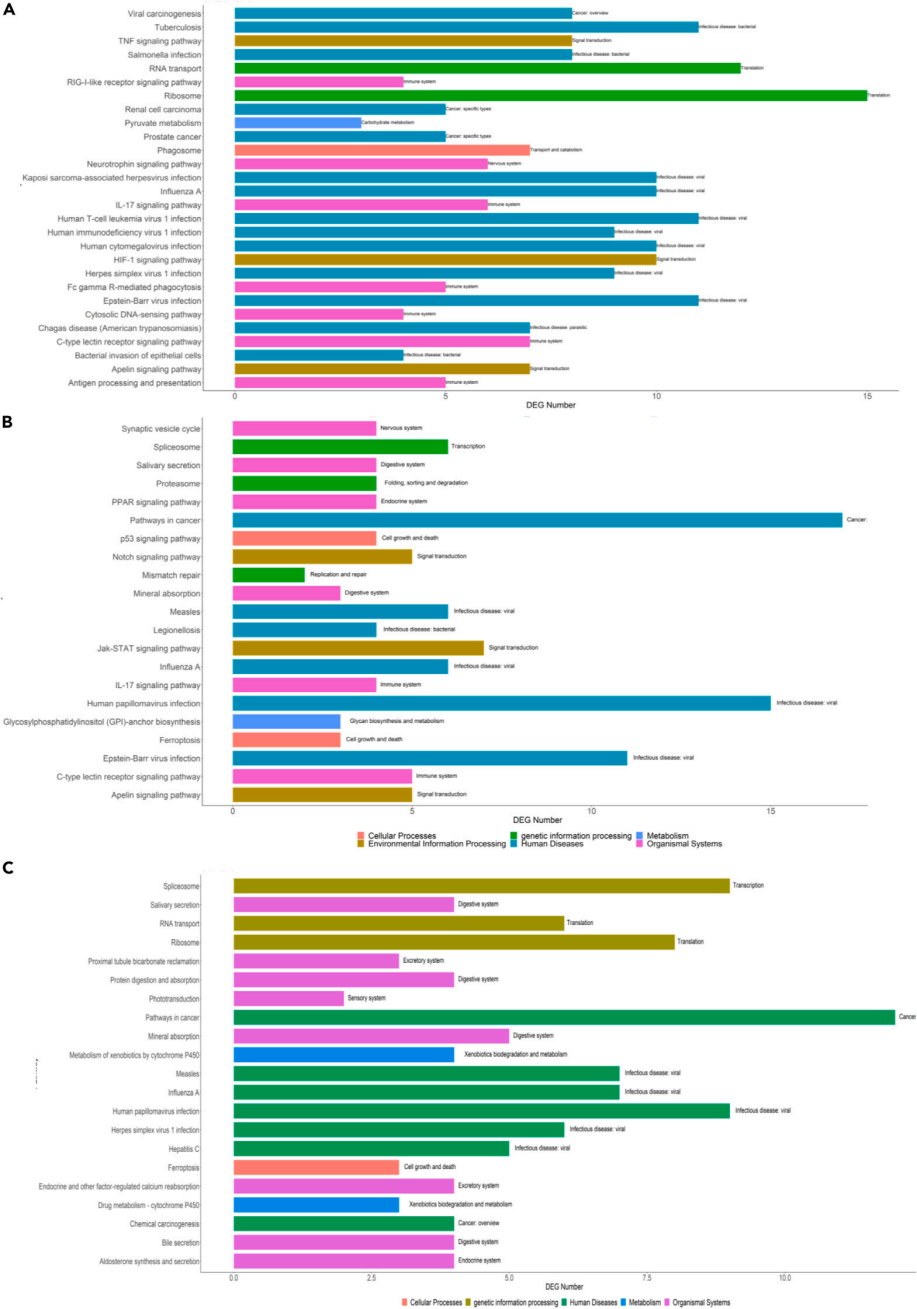
KEGG pathway enrichment analysis (A–C) KEGG functional categories characteristics of LPS-eAECs (A), LPS-mAECs (B), and both LPS-stimulated cell populations in comparison (C). The Y axis represents the KEGG functional categories, while the X axis represents the number of DEGs. The KEGG subcategories are indicated at the end of each bar. See also supplementary information: Figure S1, Data S1, from Tables S1, S2, S3, S4, and S5.

The Kyoto Encyclopedia of Genes and Genomes (KEGG)-based pathway enrichment analysis displayed 28 significantly enriched pathways in LPS-eAECs and 21 enriched pathways in LPS-mAECs, and 21 enriched pathways resulted from the comparison of the two stimulated cell populations (Table S4; Figure 1). KEGG analysis highlighted that the Influenza A pathway was the unique shared pathway among all conditions (Figure 1). Furthermore, LPS was related to the enrichment of immune system (IL-17 signaling and C-type lectin receptor signaling), infectious disease (Epstein-Barr virus infection), and signal transduction (Apelin signaling) pathways, independently from cell phenotype. However, even if eAECs and mAECs cell populations exhibited the enrichment of common signaling pathways, genes subtending those signaling cascades were different (Table S5). In LPS-eAECs stimulated cells, this response came with the enrichment of the antigen processing and presentation pathway, the RIG-1 receptor signaling pathway, FC-gamma-mediated phagocytosis, the neurotrophin signaling pathway, and the cytosolic DNA-sensing pathway, suggesting the existence of cooperative mechanisms actuated by these cells to defense against inflammatory insults.

### Gene-gene interaction network analysis reveals different controller genes

Using the data of KEGG pathways analysis, three gene-gene networks were built: LPS-eAECs network (network 1), LPS-mAECs (network 2) and a third network based on the LPS-mAECs vs. LPS-eAECs pairwise comparison (network 3). More in detail, network 1 consisted of 1,523 nodes and 7,880 edges with 14 connected components; network 2 consisted of 1,294 nodes, 11,792 edges, and 17 connected components; and network 3 consisted of 1,364 nodes and 7,625 edges grouped in 28 connected components. The topological parameters confirmed the scale-free nature of each network (Table 1).

**Table 1:** Network topological parameters analysis for each experimental group

	LPS-eAECs *vs*. eAECs	LPS-mAECs *vs*. mAECs	LPS-mAECs *vs*. LPS-eAECs
Parameters			
Number of nodes	1,523	1,294	1,364
Number of edges	7,880	11,792	7,625
Clustering coefficient	0.179	0.057	0.224
Connected component	14	17	28
Network diameter	16	18	25
Shorts paths	507,809 (21%)	324,938 (19%)	203,138 (10%)
Characteristic path length	5,949	5,992	7,833
Avg. number of neighbors	9,190	17,238	9,702
Node degree distribution (in degree/out degree)			
-γ	−1.615/-1.157	−0.965/-1.080	−1.238/-1.127
R	0.715/0.891	0.930/0.768	0.711/0.887
R2	0.771/0.705	0.528/0.542	0.577/0.646
Clustering coefficient vs. node degree			
-γ	−0.653	−0.981	−0.159
R	0.227	0.503	−0.033
R2	0.257	0.304	0.009

The table displays topological parameters computed, the node degree distribution, and correlation of node degree with clustering coefficient in the network. See also Table S6.

Network 1 analysis identified 115 bottlenecks and 114 hubs (Table S6). Among them, kernel density estimation (KDE) analysis identified 4 local hubs: T cell receptor β chain V region (TRB), class II major histocompatibility complex transactivator (CIITA), T cell receptor beta variable 7-9 (TRBV7-9), and CD74 molecule (CD74), all genes involved in the adaptive immune response mechanism. Network 2 analysis identified 209 bottlenecks and 205 hubs (Table S6). Among the latter, 4 members of the signal transducer and activator of transcription (STATs) family are recognized as local hubs by KDE analysis: STAT6, STAT3, STAT5A, and STAT5B. Finally, Network 3 analysis identified 94 bottlenecks and 93 hubs (Table S6). Only one gene of those was identified as a local hub by KDE analysis, namely, the nuclear factor erythroid 2 like 2 (NFE2L2), which plays a pleiotropic role in the regulation of inflammation, autophagy, metabolism, and immune response mechanisms.

**Figure 2:**
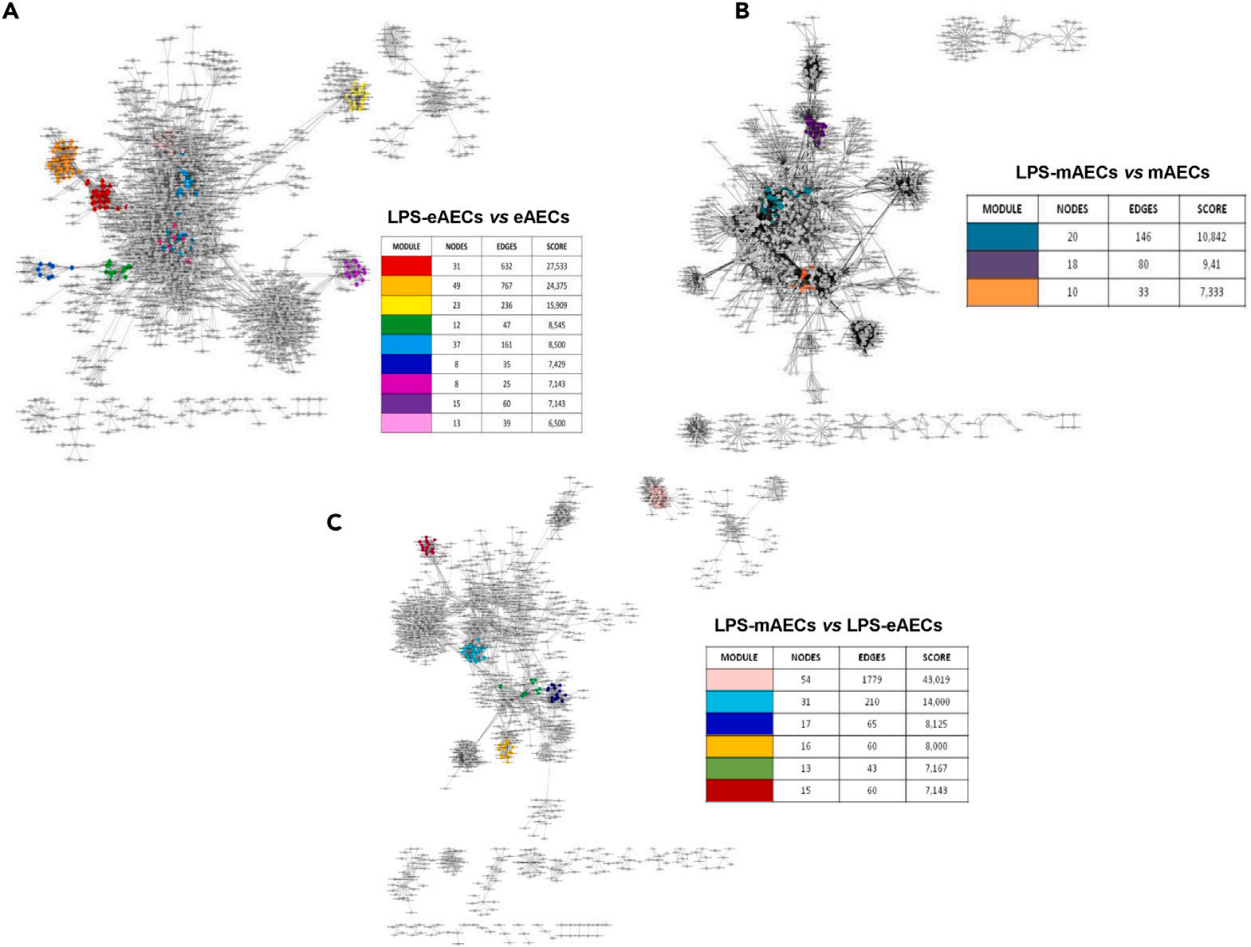
The three gene-regulatory networks with their annotated modules identified by MCODE Cytoscape plugin and annotated by ClueGO (A) LPS-eAECs vs. eAECs network, 9 modules: red (positive regulation of myeloid leukocyte differentiation), orange (antigen processing and presentation of peptide antigen via MHC class I), yellow (structural constituent of cytoskeleton), green (malic enzyme activity), pale blue (cellular response to reactive oxygen species), blue (acetyl CoA biosynthetic process), fuchsia (positive regulation of NIK/NF-kappa B signaling), purple (translation initiation factor activity), pink (protein tyrosine kinase binding). (B) LPS-mAECs vs. mAECs network, 3 modules: navy (receptor signaling pathway via JAK/STAT), dark purple (regulation of Notch signaling pathway), orange (regulation of synaptic vesicles). (C) LPS-mAECs vs. LPS-eAECs network, 6 modules: pink (glutathione metabolic process), pale blue (positive regulation of production of molecular mediator of immune response), blue (regulation of transcription from RNA polymerase II promoter in response to stress), dark orange (cardiac septum development), olive (translation initiation factor activity), dark red (synaptic vesicle exocytosis). See also Table S7.

Moreover, we analyzed the networks by means of the MCODE algorithm to identify different functional modules, which we further annotated in proper biological processes GO category by using ClueGO plugin. Network 1 was characterized by 9 functional modules (Figure 2) mainly associated with survival and immune stress response (fuchsia, pale blue, pink, green, and yellow modules) and antigen processing immune response processes (red and orange modules).

Modules related to RNA transport (purple) and metabolism (blue) were also identified (Table S7). Network 2 was characterized by 3 functional modules related to protein-mediated cell signaling response involving JAK-STAT and Notch signaling pathways (navy and dark purple modules). A module with genes playing a role in the regulation of synaptic vesicle processes (orange) was also identified (Table S7). Network 3 was characterized by 6 modules associated with cell defense (pink, pale blue, and blue modules). Modules related to infection response such as cardiac septum development, vesicle, and RNA trafficking processes (dark orange, dark red, and olive) were also identified (Table S7).

### Candidate *driver genes* managing LPS response

**Figure 3:**
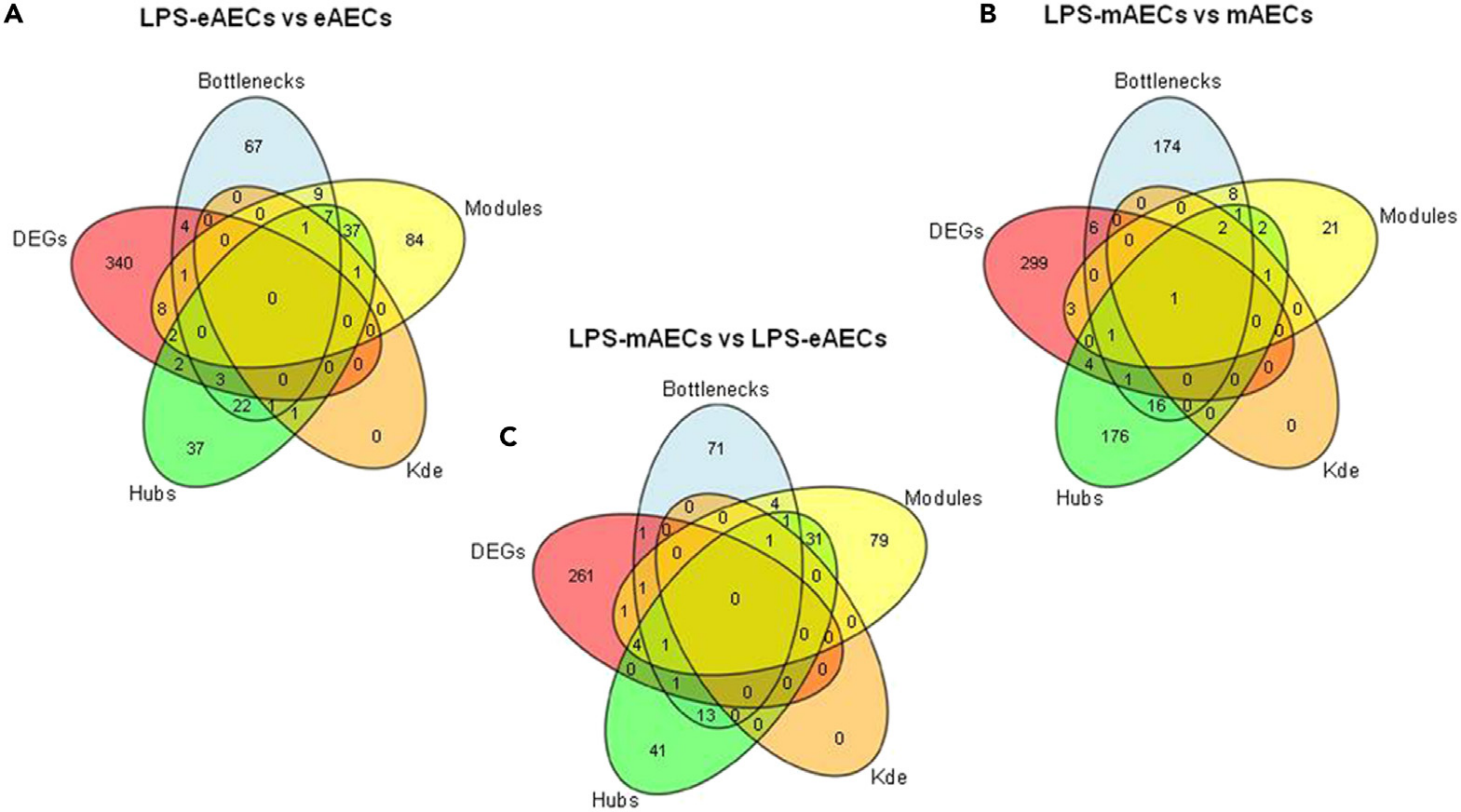
Venn diagram analysis Venn diagram reporting integration of topological and differential expression analyses related respectively to the LPS eAECs vs. eAECs (A), LPS mAECs vs. mAECs (B) and LPS mAECs vs. LPS eAECs (C) pairwise comparisons. See also Table S8.

To identify those genes that could be considered as potential “drivers” of the immune response induced by LPS response, we performed an integration of results from differential expression, topological network analysis, and module analysis (Figure 3; Table S8).

More in detail, in LPS-eAECs 34 genes displayed both hub and bottleneck node properties (Figure 3). In this category, RAN member RAN oncogene family (RAN), ras homolog family member A (RHOA), and eukaryotic translation initiation factor 2 subunit alpha (EIF2S1) also resulted to be differentially expressed (DEG) (Table S1). Two additional immune-related genes, T cell receptor β chain V region (TBR) and class II major histocompatibility complex transactivator (CIITA), also showed characteristics of local hubs.

The network analysis of the LPS-mAECs recognized 22 genes with hub-bottleneck properties. Among them, interleukin 6 (IL-6) and signal transducer and activator of transcription 2 (STAT2) also showed a DEG feature, whereas a role of local hub was also assigned to signal transducer and activator of transcription 5 (STAT5A) and signal transducer and activator of transcription 3 (STAT3) genes.

In addition, the comparative analysis between the two LPS-exposed cell populations networks highlighted 16 genes with hub-bottleneck features. Among them, STAT2 and epoxide hydrolase 1 (EPHX1) also displayed very high differential expression. Furthermore, KDE analysis identified within the hub-bottleneck category the nuclear factor erythroid 2 like 2 (NFE2L2) gene showing local hub features. These findings were summarized in Table 2.

**Table 2:** Driver genes in LPS-stimulated AECs

Gene	Gene Description	Functional annotation	Topological features			Expression features	
			Hub	Bottleneck	Local hub	DEG	Significance
RAN	RAN member RAN oncogene family	*Role in host immune response; also involved in tumorigenesis*^56^	X	X		X	X
RHOA	Ras homolog family member A	*Regulator of innate and adaptive immunity; frequently deleted in several cancer types*^57^^,^^58^	X	X		X	X
EIF2S1	Eukaryotic translation initiation factor 2 subunit alpha	*Hallmark of immunogenic cell death; also linked to neurodegenerative disorders*^59^^,^^60^	X	X		X	X
TRBV	T cell receptor β chain	*Associated with the host immune response and regenerative proliferation of liver; clonal expansion correlates with IBD disease severity*^61^^,^^62^	X	X	X		
CIITA	Class II major histocompatibility complex transactivator	*Involved in the immune rejection associated with allogeneic stem cell therapy in the heart; gene fusion has been linked to lymphoid cancers*^63^^,^^64^	X	X	X		
IL-6	Interleukin 6	*Crucial role in inflammation, immunity, allograft rejection, and cancer*^65^^,^^66^	X	X		X	X
STAT2	Signal transducer and activator of transcription 2	*Crucial role in infection, protective immunity, and immunodeficiencies*^67^^,^^68^	X	X		X	X
STAT6	Signal transducer and activator of transcription 6	*Crucial role in infection, protective immunity, and immunodeficiencies*^67^^,^^68^	X	X	X	X	X
STAT5A	Signal transducer and activator of transcription 5A	*Crucial role in infection, protective immunity, and immunodeficiencies*^67^^,^^68^	X	X	X		
STAT3	Signal transducer and activator of transcription 3	*Crucial role in infection, protective immunity, and immunodeficiencies*^67^^,^^68^	X	X	X		
EPHX1	Epoxide hydrolase 1	*Detoxification and bioactivation of endogenous epoxides regulating metabolic homeostasis; role in proteinuria-induced EMT of renal epithelial cells; mutations and gene variants have been associated with susceptibility to several inflammatory diseases*^46^^,^^69^^,^^70^	X	X		X	X
NFE2L2	Nuclear factor, erythroid 2 like 2	*Detoxification, metabolism, and inflammation processes; involved in EMT*^71^^,^^72^	X		X		
GSTA4	Glutathione S-transferase alpha 4	*NFE2L2-regulated transcript involved in detoxification and metabolism of toxic and carcinogenic compounds; upregulated in inflammation models of colorectal cancer where it mediates chemoresistance*^73^^,^^74^	X	X			
GSTP1	Glutathione S-transferase Pi 1	*Detoxification, metabolism, and inflammation processes; involved in EMT in cancer*^75^^,^^76^^,^^77^	X	X			

The main biological functions of each driver gene have been reported. The table also shows the topological and the expression features identified from computational analyses. See also Figure S2.

Furthermore, the differential expression of some genes with specific topological features or exhibiting levels of fold change was validated by qRT-PCR (Figure S2). Briefly, in LPS-eAECs two local hubs (CIITA and CREB) were down-expressed while the B2M gene confirmed its over-expression. Instead, in the LPS-mAECs real-time qPCR confirmed the over-expression of the two bottleneck genes DDX58 and IL-6 (the latter also showed hub properties). Similarly, the NFLE2L gene that performed as hub in the comparison of the two LPS-stimulated populations was confirmed as over-expressed in the mAECs with respect to the eAECs (Figure S2).

### CIITA and Nrf2 regulate the response to LPS of AECs pre- and post-EMT

**Figure 4:**
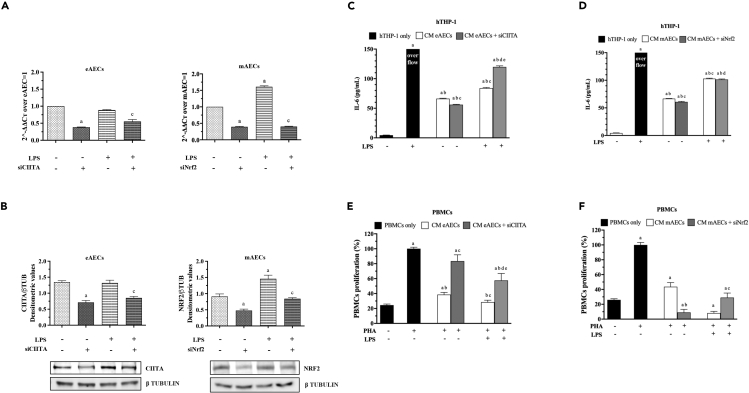
CIITA and Nrf2 regulate eAECs and mAECs immunoresponses (A and B) Downregulation of CIITA and Nrf2 at mRNA (A) and protein (B) levels, respectively, in eAECs and mAECs was confirmed by real-time qPCR and western blot quantification. Results from gene and protein expression analyses (A and B) are the mean ± SD, from n = 3 independent experiments. The significance of data related to eAECs (p < 0.05) was statistically analyzed vs. eAECs and indicated with (a), vs. siCIITA with (b), and vs. LPS with (c), respectively. The significance of data related to mAECs (p < 0.05) was statistically analyzed vs. mAECs and indicated with (a), vs. siNrf2 with (b), and vs. LPS with (C), respectively. Student’s t-tail test was used. (C–F) CM derived from eAECs and mAECs was used to test the effect of siCIITA and siNrf2 on IL-6 secretion by THP-1 (C and E) and proliferation attitude of PBMCs (D-F). Quantification of IL-6 secretion by THP-1 following incubation with CM derived from eAECs (C) and mAECs (E). Results from immunomodulation analyses with THP-1 are the mean ± SD, from n = 3 independent experiments. The significance of data (p < 0.05) was statistically analyzed vs. THP-1 and indicated with (a), vs. THP-1 + LPS with (b), vs. NTC with (c), vs. siCIITA or siNrf2 with (d), and vs. NTC + LPS with (e), respectively. PBMCs proliferation rate upon incubation with eAECs (D) and mAECs (F). Results from immunomodulation analyses with PBMCs are the mean ± SD, from n = 3 independent experiments. The significance of data (p < 0.05) was statistically analyzed vs. PBMC and indicated with (a), vs. PHA 2.5 ug/ml with (b), vs. NTC with (c), vs. siCIITA or siNrf2 with (d), and vs. NTC + LPS with (e), respectively. Student’s t-tail test was used.

In order to confirm whether CIITA and Nrf2 play key roles in the regulation of eAECs and mAECs immune response, respectively, transient gene depletion of these two factors by using specific siRNAs has been performed. The downregulation of either CIITA or Nrf2 was confirmed at mRNA (Figure 4A) and protein (Figure 4B) levels up to 48 h.

Thereafter, siRNA-CIITA eAECs and siRNA-Nrf2 mAECs were exposed to LPS inflammatory stimulus for 1 h and serum-free conditioned media (CMs) were collected after 24 h. We investigated the immunomodulatory effect of CM derived from silenced eAECs and mAECs, respectively, through two different immune tests (Figures 4C–4F). Our data showed that eAECs and mAECs have two divergent immune behaviors; while the eAECs are mainly anti-inflammatory, the mAECs possess both anti- and pro-inflammatory properties.

In detail, the pro-inflammatory ability of the cells was determined by subjecting THP-1 macrophages to the collected CMs to assess the IL-6 production, used as a marker of their activation. In detail, CM derived from LPS-stimulated eAECs induced a significant increase of IL-6 production by THP-1 macrophages with respect to CM derived from non-stimulated eAECs (Figure 4C). Instead, the silencing of CIITA in eAECs significantly decreased IL-6 release with respect to non-silenced cells (Figure 4C). Interestingly, the transient depletion of CIITA in LPS-stimulated eAECs induced the highest IL-6 production, thus suggesting the anti-inflammatory role of this gene (Figure 4C). A similar trend of IL-6 production was recorded for CM derived from mAECs except for silenced mAECs stimulated with LPS (Figure 4D). In particular, THP-1 macrophages showed a significant increase of IL-6 production when subjected to CM derived from LPS-stimulated mAECs (Figure 4D). As with silenced eAECs, the transient depletion of Nrf2 in mAECs significantly decreased this release with respect to non-silenced cells (Figure 4D). Conversely, the CM derived from silenced mAECs did not impair the ability of mAECs to activate THP-1 macrophages when stimulated with LPS (Figure 4D).

Furthermore, the CMs collected from both eAECs and mAECs were added to phytohemagglutinin (PHA)-stimulated peripheral blood mononuclear cells (PBMCs) to evaluate CM effect on PBMCs proliferation. To this regard, the inhibition of PBMCs proliferation was used as an index of CM anti-inflammatory abilities. The obtained results demonstrated that CM derived from eAECs reduced PHA-stimulated PBMCs proliferation of 65% (Figure 4E). Moreover, CM derived from LPS-stimulated eAECs induced a significant decrease of PBMCs proliferation with respect to non-stimulated cells (Figure 4E). Of note, CIITA silencing dramatically impaired the eAECs-derived CM inhibitory effect on PBMCs proliferation with respect to non-silenced cells (Figure 4E). This trend was consistently maintained in the presence of LPS stimulation (Figure 4E). As with eAECs, CM derived from mAECs reduced PHA-stimulated PBMCs proliferation of 55% (Figure 4E). Besides, CM derived from LPS-stimulated mAECs induced a massive reduction of PBMCs proliferation with respect to non-stimulated cells (Figure 4E). Surprisingly, CM derived from silenced mAECs exhibited a similar inhibitory effect on PBMCs proliferation as recorded for CM collected from LPS-stimulated mAECs (Figure 4E). Finally, CM collected from silenced mAECs stimulated with LPS did not induce a comparable inhibition as recorded for silenced cells, although PBMCs proliferation was very low (Figure 4E).

## Discussion

Here, for the first time we reported that eAECs and mAECs possessed different immune behaviors and showed divergent immunomodulatory responses during inflammation, thus demonstrating that AECs phenotype may highly affect the immune response of these stem cells. In this context, we have recently shown that both eAECs and mAECs enhanced tendon regeneration *in vivo* by displaying distinct immunomodulatory profiles.^28^ In detail, the ability of these cells to convey the shift from pro-inflammatory to pro-regenerative responses in the host tissue differed in terms of macrophages polarization, cellularity, and blood vessel remodeling.^28^ Based on these premises, to further understand the molecular mechanisms underlying the cells’ immune response, RNA-seq was used to compare the whole transcriptomics profiles in eAECs and mAECs. Subsequently, the adoption of network theory allowed identification of two different immune behaviors: eAECs mainly participate in the immune response by upregulating antigen-presenting circuit; on the other hand, mAECs mainly responded by upregulating cytokine and soluble mediators. Analysis of the gene involved in these two responses led to the identification of CIITA and Nrf2 as candidate driver genes, respectively, for eAECs and mAECs. Finally, the transient depletion of these genes in eAECs and mAECs demonstrated that they play a fundamental role for the respective immune responses: on the one hand, eAECs anti-inflammatory ability is dramatically compromised by the transient CIITA depletion. On the contrary, the contribution of mAECs to the immune system activation is highly impaired by the transient Nrf2 depletion, further suggesting that EMT may indeed represent a key event for the determination of AECs functions.

As epithelial cells, AECs play a fundamental role as a natural barrier between the organism and the environment (and fetus), developing, in turn, common strategies to regulate tolerance while preserving immunity against pathogens.^29^ Therefore, LPS stimulation of eAECs activates specific intracellular signaling belonging to different members of the TLR4/NF-κB/type I IFN pathway, identified as controllers of the interaction network. Of note, there is a group of genes endowed with antigen processing and presenting functions, such as CIITA, TRBV, rano class II histocompatibility antigen (α chain bL3-7 like and β chain like), β2-microglobulin (B2M), and RhoA. CIITA is a member of the NLR (nucleotide-binding and leucine-rich-repeat-containing) protein family and is recognized as the master regulator of MHC-II gene expression.^30^ Surprisingly, even though a master regulator of MHC-I gene has been identified (NLRC5), it was found that CIITA can indeed activate MHC-I expression, especially in cell lines like AECs^31^^,^^32^ where those antigens are negative or have very low expression.^33^^,^^34^ Moreover, eAECs participate in the immune system activation through the upregulation of MHC-II proteins and all the complex of the proteins related to the antigen presenting process.^35^^,^^36^ To this regard, here we demonstrate that the transient depletion of CIITA dramatically impairs the anti-inflammatory abilities of eAEC, thus demonstrating its pivotal role in the immune response of these cells.^37^

Conversely, the modulation of MHC genes seems not to be the primary mAECs mechanism in response to LPS. In fact, mAECs show the activation of a plethora of intracellular pathways specifically related to tyrosine kinase receptors (EGFR, PDGF, GHR, CSF3R, JAK/STATs) signaling and interleukins (IL-6, IL-12B, IL-10) production. In particular, the immune response of mAECs relies on the activation of STAT2 and IFN-stimulated genes, many of which encode proteins with antiviral, anti-proliferative, pro-apoptotic, or immunomodulatory functions.^38^^,^^39^ The upregulation of IL-6 may possibly be mediated by STAT2 itself in response to the classical NF-κB activators (e.g., LPS), as recently demonstrated.^40^ On the contrary, although STAT6 bears features of local hub, date hub, bottleneck, and DEG in mAECs, this gene is significantly downregulated. Interestingly, STAT6 is primarily involved in signaling activated by IL-4 and IL-13, which play roles in T helper 2 (Th2) differentiation, immunoglobulin class switching, macrophage activation, and MHC-II expression.^39^ This could partially explain the absent MHC-II gene modulation in mAECs. In this scenario, eAECs may act as inducer of Th2-like immune response, whereas mAECs could be more involved in triggering a Th1-like response.^41^^,^^42^^,^^43^^,^^44^

**Figure 5:**
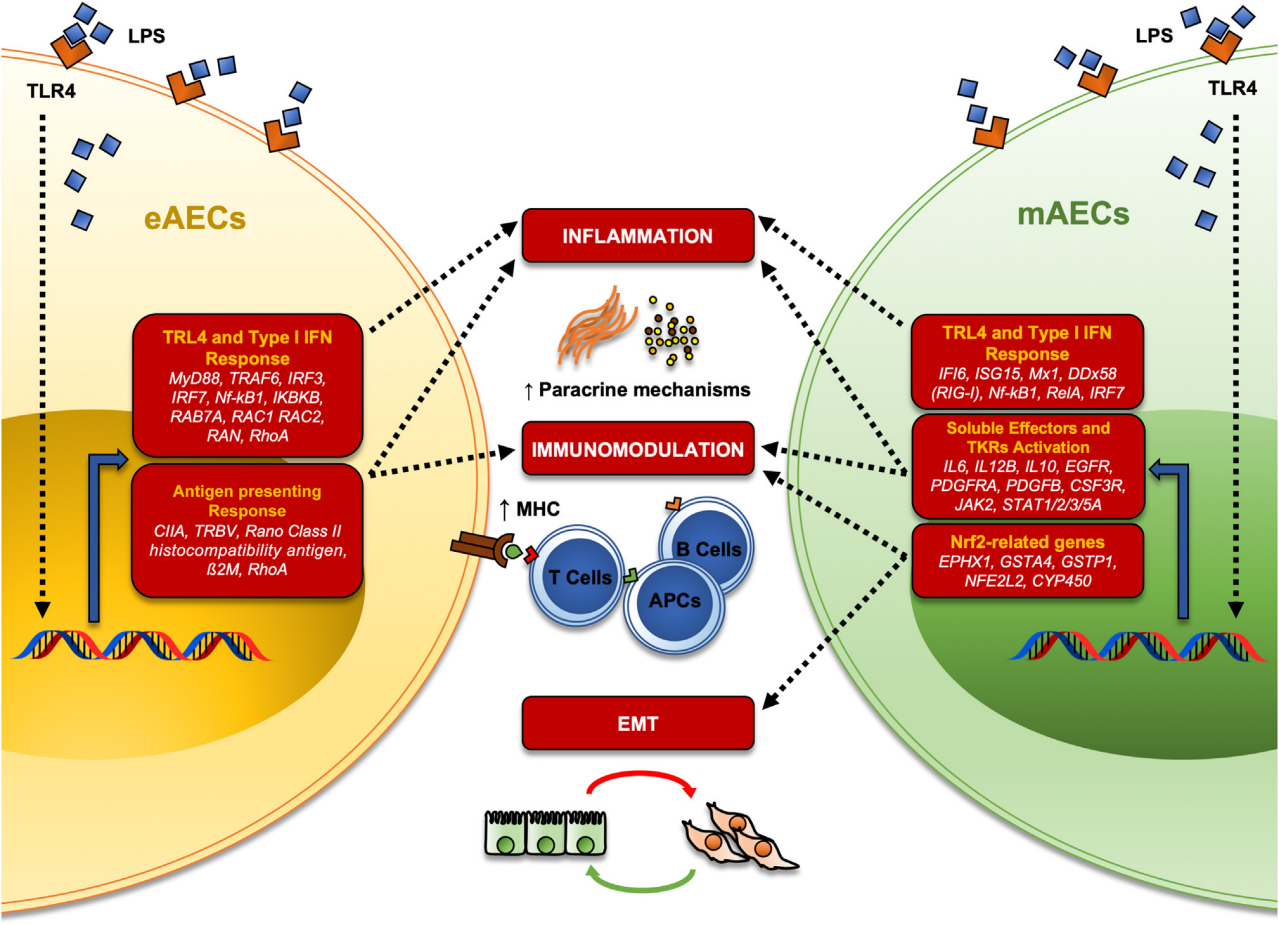
Differential gene regulation in eAECs and mAECs upon LPS stimulation Schematic representation of upregulated driver genes upon LPS stimulation in eAECs and mAECs and the related biological functions. β2M, Beta-2 microglobulin; IL, interleukin; TKR, tyrosine kinase receptor, CYP450, cytochrome P450. Figure created with Biorender.com.

However, the comparison between LPS-mAECs and LPS-eAECs points out a group of genes implicated in either EMT or immunomodulatory process (Figure 5). In particular, most of the driver genes belong to the Nrf2 pathway (e.g., EPHX1, GSTP1,and NFE2L2), a transcription factor that we previously described to be involved in EMT of AECs.^25^^,^^45^^,^^46^ Of note, mAECs immune response with respect to their epithelial counterpart (eAECs) is not strictly anti-inflammatory. To this regard, the dual role played by Nrf2 in leading either anti-inflammatory or inflammatory responses may potentially explain the results observed in mAECs.^47^ Indeed, it was extensively demonstrated that a functional cross talk exists between NF-kB and Nrf2 pathways and that can enhance or impair the inflammatory response, depending on the specific context and stimuli.^47^ Although from a functional perspective Nrf2 negatively controls the NF-κB pathway by multiple mechanisms,^48^^,^^49^^,^^50^ our results indicated that LPS-mAECs preferentially activate both pathways. Moreover, the transient depletion of Nrf2 highly enhances their anti-inflammatory property and reduces the pro-inflammatory property, thus suggesting a potential involvement in the activation of inflammatory pathways, likely depending on the activation of the NF-κB pathway, even though further experiments are needed to demonstrate this hypothesis.

All together these results point out an intriguing scenario in which EMT plays a significant role in determining the immune response not only in isolated AECs but also in whole AM, where the two cell populations coexist. An interesting speculation is related to the possibility that AECs in AM could undergo EMT/MET to facilitate inflammation resolution by exploiting alternative strategies. At the same time, the two cell populations could cooperate either in damping inflammation or in remodeling AM during pregnancy and labor. In this scenario, epithelial or mesenchymal cells of AM would undergo EMT/MET upon LPS or other environmental stimuli (e.g., oxidative stress or pathogens). Notably, several authors reported that LPS can induce EMT via JAK/STAT and NF-κB signaling,^51^^,^^52^^,^^53^^,^^54^^,^^55^ two of the most upregulated pathways in both eAECs and mAECs. However, further studies are necessary to explore this fascinating possibility and the relative consequences.

## STAR★Methods

### Key resources table

**Table:** 

REAGENT or RESOURCE	SOURCE	IDENTIFIER
**Antibodies**		

Mouse monoclonal anti-Cytokeratin 8	Abcam	Abcam Cat# ab2530; RRID:AB_303136)
Mouse monoclonal anti-Vimentin 9	Agilent	Agilent Cat# M0725; RRID:AB_10013485
Rabbit polyclonal anti-Nrf2	Novus	Novus Cat# NBP1-32822; RRID:AB_10003994
Rabbit polyclonal anti-CIITA	Biorbyt	Biorbyt Cat# orb182642; RRID:AB_1846771
Mouse monoclonal anti-Alpha Tubulin	Sigma-Aldrich	Sigma-Aldrich Cat# T5168; RRID:AB_477579
Mouse anti-rabbit IgG-HRP	Santa Cruz Biotechnology	Santa Cruz Biotechnology Cat# sc-2357; RRID:AB_628497
Mouse IgGκ-HRP	Santa Cruz Biotechnology	Santa Cruz Biotechnology Cat# sc-516102; RRID:AB_2687626
Goat anti-Mouse polyclonal IgG Cy3 conjugate	Millipore	Millipore Cat# AP124C; RRID:AB_11213281
Goat anti-Mouse polyclonal IgG H&L Alexa Fluor 488	Abcam	Abcam Cat# ab150113; RRID:AB_2576208

**Chemicals, peptides, and recombinant proteins**		

Lipopolysaccharide (LPS) from Escherichia coli O55:B5	Sigma-Aldrich	Cat# L2637
Phorbol 12-myristate 13-acetate (PMA)	Sigma-Aldrich	Cat# P1585
Phytohemagglutinin-L (PHA-L)	Invitrogen	Cat# 00-4977-93
Sodium pyruvate	Gibco	Cat# 11360-070
Penicillin-Streptomycin	Lonza	Cat# DE 17-602E
Amphotericin B	Euroclone	Cat# ECM0009D
L-Glutamine	Euroclone	Cat# ECB300D
4 pregnene-3,20-dione (P4)	Sigma-Aldrich	Cat# P8783
Ficoll-Paque PLUS	Amersham Biosciences	Cat# 18-1152-69
Fluoromount	Sigma-Aldrich	Cat# F4680
4′,6-diamidino-2-phenylindole (DAPI)	Sigma-Aldrich	Cat# D9542
Formol 4% (Paraformaldehyde)	VWR	Cat# 11699404
Triton X-100	Sigma -Aldrich	Cat# T8787
Phosphate Buffered Saline (PBS)	Sigma-Aldrich	Cat# P3813
Bovine Serum Albumin (BSA)	Sigma-Aldrich	Cat# P3813
TransIT-X2® Dynamic Delivery System	Mirus	Cat# MIR-6000
Tetro Reverse Transcriptase	Bioline	Cat# BIO-65050
Phosphatase Inhibitor	Sigma-Aldrich	Cat# P5726
Protease Inhibitor Cocktails	Sigma-Aldrich	Cat# P8340
Quick Start™ Bradford 1x Dye Reagent	Bio-Rad Laboratories	Cat# 5000205
4X Laemmli Sample buffer	Bio-Rad Laboratories	Cat# 1610747
ClarityMax ECL reagents	Bio-Rad Laboratories	Cat# 1705062
Oligo-dT beads	Illumina	Cat# 20020595

**Critical commercial assays**		

Human IL-6 Uncoated ELISA Kit assay	Invitrogen	Cat# 88-7066-88
CellTiter96 Aqueous One Solution Cell Proliferation Assay	Promega	Cat# G3582
RNeasy Mini Kit	QIAGEN	Cat# 74106
Dynabeads™ mRNA Purification Kit	Invitrogen	Cat# 61006
Direct-zol RNA kit	Zymo research	Cat# R2071
SensiFAST SYBR Lo-ROX kit	Bioline	Cat# BIO-94050
TruSeq Stranded mRNA Library Prep Kit	Illumina	Cat# 20020594

**Experimental models: Cell lines**		

Ovine: Amniotic Epithelial Cells (AECS)	Amniotic membrane of female sheep	N/A
Ovine: Peripheral blood mononuclear cells (PBMCs)	Peripheral ovine blood	N/A
Human: THP-1 cell line	ATCC	TIB-202

**Oligonucleotides**		

Custom ON-TARGETplus SMARTpool targeting Nrf2, See Table S9	Dharmacon	Cat# FE52L001000005
Custom ON-TARGETplus SMARTpool targeting CIITA, See Table S9	Dharmacon	Cat# FE51L0010000050
ON-TARGETplus Non-targeting Pool (NTC), See Table S9	Dharmacon	Cat# FE5D0018101005
Primers for qRT-PCR, See Table S10	This paper	N/A
OligodT primers	Bioline	Cat# BIO-38029

**Software and algorithms**		

Zen 3.4 Blue edition software	Zeiss	https://www.micro-shop.zeiss.com/en/us/softwarefinder/software-categories/zen-blue/
Cytoscape 3.7.1 software	Shannon et al.^87^	N/A
Cytoscape plugin CytoHubba	Chin et al.^90^	N/A
Cytoscape plugin Molecular Complex Detection (MCODE)	Bader et al.^91^	N/A
Trimmomatic software	Bolger et al.^79^	https://bioinformaticshome.com/tools/rna-seq/descriptions/Trimmomatic.html#gsc.tab=0
Kallisto aligner software	Bray et al.^27^	https://github.com/pachterlab/kallisto
ImageJ 1.53k software	This paper	https://ImageJ.nih.gov/ij/
EdgeR package	McCarthy et al.^81^	https://bioconductor.org/packages/release/bioc/html/edgeR.html
Rgraphviz R-package	This paper	https://www.bioconductor.org/packages/release/bioc/html/Rgraphviz.html
GO R-package	This paper	https://bioconductor.org/packages/release/bioc/html/topGO.html
ClusterProfiler package	Yu et al.^84^	https://guangchuangyu.github.io/software/clusterProfiler/
KEGGgraph R-package	Yu et al.^86^	https://www.bioconductor.org/packages/release/bioc/html/KEGGgraph.html
FastQC tool	This paper	https://www.bioinformatics.babraham.ac.uk/projects/fastqc
BioMart tool	Smedley et al.^82^	https://bioconductor.org/packages/biomaRt/

**Deposited Data**		

Accession code for RNA-Seq	NCBI Sequence Read Archive (SRA)	BioprojectID: PRJNA989111

**Other**		

Fetal Bovine Serum (FBS)	Gibco	Cat# 10270-106
MEM Eagle Alpha	Euroclone	Cat# ECM0850L
RPMI 1640 medium containing glutamine	Gibco	Cat# 61870-010
Gene Ontology (GO)	This paper	http://geneontology.org
Kyoto Encyclopedia of Genes and Genomes (KEGG)	This paper	https://www.genome.jp/kegg/
Ensembl 99 database	Hunt et al.^80^	http://jan2020.archive.ensembl.org/index.html
ReviGO web server	Supek et al.^83^	http://revigo.irb.hr/

### Resource availability

#### Lead contact

Further information and requests for resources and reagents should be directed to and will be fulfilled by the lead contact, Alessia Peserico (apeserico@unite.it).

#### Materials availability

All data reported in this paper will be shared by the lead contact upon request.

### Experimental model and study participant details

#### Cell culture

AECs were isolated from the amniotic membrane of female sheep at mid-pregnancy according to Canciello et al. (2017; 2018). In particular, we have collected AECs from three different fetus (two males and one female) ranging between 20 and 25 cm of length. AECs were mantained in MEM Eagle Alpha (ECM0850L, Euroclone) supplemented with 10% fetal bovine serum (FBS; #10270-106, Gibco), 100 IU/mL penicillin-streptomycin (DE 17-602E, Lonza), 100 IU/mL Amphotericin B (ECM0009D, Euroclone) and 100 IU/mL L-Glutamine (ECB300D, Euroclone). eAECs and mAECs were obtained respectively by culturing AECs for three passages in presence and absence of 25 μM progesterone (4 pregnene-3,20-dione, P4; P8783, Sigma-Aldrich). Both cell types were subsequently exposed to 1 μg/mL Lipopolysaccharide (LPS; L2637, Sigma-Aldrich) for 24 h.

The human THP-1 cell line was obtained from ATCC. THP-1 cells were maintained in RPMI 1640 medium containing glutamine (#61870-010, Gibco) and supplemented with 10% FBS (#10270-106, Gibco), 1 mM sodium pyruvate (#11360-070, Gibco) and 100 IU/mL penicillin-streptomycin (DE 17-602E, Lonza). THP-1 cells (2–3 × 10ˆ^5^ cells/mL) were differentiated using 100 nM phorbol 12-myristate 13-acetate (PMA; P1585, Sigma-Aldrich) for 2 days.^78^

Peripheral blood mononuclear cells (PBMCs) were obtained by density gradient centrifugation with Ficoll-Paque PLUS (#18-1152-69, Amersham Bisciences) of 15 mL peripheral ovine blood following manifacturer’s instruction. 2.5x10ˆ^5^ PBMCs were plated in 96-well plates with MEM Eagle Alpha (ECM0850L, Euroclone), supplemented with 5% FBS (#10270-106, Gibco) and activated by adding phytohemagglutinin-L (PHA-L; #00-4977-93 Invitrogen) at a final concentration of 2.5 μg/mL.

AECs and PBMCs cultures were performed in a 38.5°C and 5% CO2 incubator. THP-1 cell culture was carried out in a 37°C and 5% CO2 incubator.

### Method details

#### Chemicals

LPS from Escherichia coli 055:B5 (L2637) and PMA (P1585) and P4 (P8783) were were purchased from Sigma-Aldrich. PHA (#00-4977-93) was purchased from Invitrogen.

#### Immunofluorescence

Cells were growth on glass coverslips, treated as indicated in the cell culture section and then fixed in 4% paraformaldehyde (#11699404, VWR) for 10 min and permeabilized with 0.2% (v/v) Triton X-100 (T8787, Sigma -Aldrich) in 1X Phosphate Buffered Saline (PBS; P3813, Sigma-Aldrich) for 10 min. Glass coverslips were then blocked in % (w/v) Bovine Serum Albumin (BSA; A3059, Sigma-Aldrich) in 1X PBS for 1 h at room temperature (RT) and stained overnight at 4°C with the following primary antibodies: anti-Cytokeratin-8 (1:200) (ab2530, Abcam) and anti-Vimentin (1:200) (M0725, Agilent DAKO). Cy3 (AP124C, Millipore) or Alexa Fluor 488 (ab150113, Abcam) conjugated anti-mouse secondary antibodies were diluted 1:200 in 1% (w/v) BSA/PBS and incubated on glass coverslips for 40 min at RT. Nuclear counterstaining was obtained with 4′,6-diamidino-2-phenylindole (DAPI; D9542 Sigma-Aldrich) diluited 1:5000 in 1X PBS. Coverslips were mounted with Fluoromount (F4680, Sigma-Aldrich) and cell samples analyzed using an Axioskop 2 Plus incident light fluorescence microscope (Zeiss) equipped with a CCD camera (Axiovision Cam 208color, Zeiss), with a resolution of 1300 × 1030 pixels, configured for fluorescence microscopy, and interfaced to a computer workstation, provided with an interactive and automatic image analyser (Zen 3.4 Blue edition Software, Zeiss). Digital images were acquired using standard filters setup for Cy3, Alexa Fluor 488 or DAPI.

#### RNA extraction and library construction

RNA extraction was performed on each biological sample using RNeasy Mini Kit (#74106, QIAGEN) according to the manufacturer’s instruction. RNA was quantified and checked for RNA integrity number (RIN) using the Agilent 2100 Bioanalyzer imposing a minimum value of 8.0 for processing. mRNA (poly-A) was isolated using oligo-dT beads (#20020595, Illumina) then purified using Dynabeads mRNA Purification Kit (#61006, Invitrogen). Libraries were prepared using the TruSeq Stranded mRNA Library Prep Kit (#20020594, Illumina) according to the manufacturer’s instructions.

#### RNA sequencing and quality control

The sequencing reaction has been performed on an Illumina HiSeq 2500 platform in single-end for 75 cycles ensuring at least 30 million of reads per sample.^79^ To assess the quality of sequences, raw reads were subjected to quality check using fastQC tool (https://www.bioinformatics.babraham.ac.uk/projects/fastqc) and then trimmed by Trimmomatic software (https://bioinformaticshome.com/tools/rna-seq/descriptions/Trimmomatic.html#gsc.tab=0),^80^ imposing a minimum quality per base of 24 and a minimum length of reads equal to 36 bases (ILLUMINACLIP:TruSeq3-SE:2:30:10 LEADING:3 TRAILING:3 SLIDINGWINDOW:4:24 MINLEN:36). Only those transcripts containing assembled regions were considered and only the longest alternative splicing transcripts from a specific gene were retained. The analysis was then conducted at gene level by assigning the corresponding gene to each transcript.

#### Differential expression analysis

Clean reads were pseudo-aligned against the Oviaries3.1 transcriptome downloaded from Ensembl 99 database (http://jan2020.archive.ensembl.org/index.html),^81^ using the Kallisto aligner (https://github.com/pachterlab/kallisto)^27^ with default parameters for single-end reads to allow paired sample analysis. Estimated count tables were extracted from the output and used to feed the edgeR package (https://bioconductor.org/packages/release/bioc/html/edgeR.html)^82^ using the fit model for paired samples. Reference transcripts were downloaded via Biomart (https://bioconductor.org/packages/biomaRt/).^83^ For this experiment, expression values with a corrected p value (FDR<0.05) lower than 0.05 were retained. Only those genes showing a |Log2FC| > 1 were considered as differentially expressed (DEG).

#### Gene ontology and KEGG pathways enrichment

For each gene, the annotation of GO terms for the three categories (http://geneontology.org) and the Kyoto Encyclopedia of Genes and Genomes (KEGG; https://www.genome.jp/kegg/) has been downloaded from the Ensembl database using the BioMart tool (https://bioconductor.org/packages/biomaRt/).^83^ DEGs were independently annotated in the three main GO ontologies (Biological process, Cellular component, and Molecular function) and successively it was carried out the enrichment analysis using the Fisher’s test, implemented in the top GO R-package (https://bioconductor.org/packages/release/bioc/html/topGO.html) and Rgraphviz R-packages (https://www.bioconductor.org/packages/release/bioc/html/Rgraphviz.html). The enriched GO terms were also visualized by ReviGO (http://revigo.irb.hr/).^84^ Only those GO terms having an adjusted p value (q-value) ≤ 0.01 were significantly enriched. The differentially expressed genes were also mapped to KEGG database using the clusterProfiler package (https://guangchuangyu.github.io/software/clusterProfiler/)^85^ to identify biological pathways enriched in each compared condition. The KEGG pathways enrichment analysis was carried out using a hypergeometric test implemented in the edgeR package (https://bioconductor.org/packages/release/bioc/html/edgeR.html).^86^ The pathways with a q-value ≤0.05 were recognized to be significantly enriched.

#### Gene act network building and analysis

The integration of differential expression with enriched KEGG pathways led to the building of a characteristic gene-act-network. Network was built using the KEGGgraph R-package (https://www.bioconductor.org/packages/release/bioc/html/KEGGgraph.html)^87^; then to reduce complexity, the chemical compounds were removed from the final graph. For visualization, main component selection and topological features extraction Cytoscape 3.7.1 software was used.^88^ The hubs were identified as previously described,^89^^,^^90^ by using the following equation: ND > μ + σ, where ND is the node degree, μ is the mean node degree, and σ is the node degree standard deviation. The identification of bottlenecks was carried out using the Cytoscape (Cytoscape Consortuim, http://www.cytoscapeconsortium.org/) plugin CytoHubba. It implements the following algorithm for bottleneck calculation: Ts is the shortest path tree rooted at node s. BN(v) = Σs∈V ps(v), where ps(v) = 1 if more than |V(Ts)|/4 paths from node s to other nodes in Ts meet at the vertex v; otherwise, ps(v) = 0 ^91^.

#### Modules extraction

The Cytoscape plugin Molecular Complex Detection (MCODE)^92^ was used to extract and analyze clusters of highly interconnected nodes (modules) within the network. The significant modules with k-core >5 and nodes degree >5 were further selected for GO BP analysis to evaluate the biological processes where these modules involved in, which was performed using the Cytoscape ClueGO plugin.

#### Transient silencing of Nrf2 and CIITA with siRNA and conditioned media collection

Transient gene depletion was performed by siRNAs using 50 nM ON-TARGET plus, small interfering RNA (siRNA) targeting Nrf2 (FE52L001000005, Dharmacon), CIITA (FE51L0010000050, Dharmacon) and Non-Targeting Control (hereafter referred as NTC) (FE5D0018101005, Dharmacon) and the TransIT-X2 Dynamic Delivery System (MIR-6000, Mirus) reagents, respectively, according to the manufacturers’ recommendation. In detail, to improve the transfection, cells were grown until they reached 50–60% of confluency and then transient gene depletion was performed by using specific siRNAs. After 48 h of transfection cells were starved in serum free medium for 4 h and then they were immune activated with 1 μg/mL LPS for 1 h. Afterward, the medium was replaced with fresh serum free medium that was collected after 24 h (hereafter referred as conditioned medium; CM) for immune assays inclucing THP-1 macrophages IL-6 secretion and PBMCs proliferation assay. Cell pellets were collected to confirm transient gene depletion by Real-time qPCR and Western Blot. Target sequences of siRNAs have been reported in Table S9.

#### Real time qPCR

Total RNA was extracted with Direct-zol RNA kit (R2071, Zymo research) following the manufacturer’s instructions. 1 μg of total RNA was retrotranscribed using oligodT primers (BIO-38029, Bioline) and Tetro Reverse Transcriptase (BIO-65050, Bioline), following the manufacturer’s instructions. qPCRs were carried out in triplicate using the SensiFAST SYBR Lo-ROX kit (BIO-94050, Bioline) on a 7500 Fast Real-Time PCR System (Thermo Fisher Scientific), according to the manufacturer’s instructions. The following PCR conditions were used for all experiments: 95°C for 10 min, followed by 40 cycles at 95°C for 10 s and 60°C for 30 s. Relative quantification was performed by using the ΔΔCt method. GAPDH (Glyceraldehyde 3-phosphate dehydrogenase) and YWHAZ (14-3-3 protein zeta/delta) were selected amongst housekeeping genes for gene quantification. Expression profiles were similar with both reference genes. Sequences of primers and conditions used in real-time qPCR are reported in Table S10.

#### Total protein extraction and western blotting

Total protein was extracted from each sample in lysis buffer (50 mM Tris HCl pH 8, 250 mM NaCl, 5 mM EDTA, 0.1% Triton X-100 10%) with Phosphatase Inhibitor (P5726, Sigma-Aldrich) and Protease Inhibitor Cocktails (P8340, Sigma-Aldrich) diluted according to manufacturing instruction. Cell extracts of samples were put on ice for 30 min and centrifuged at 12,000× g for 10 min at 4°C, and then protein concentration was determined by using Quick Start Bradford 1x Dye Reagent (#5000205, Bio-Rad Laboratories). Next, 30 μg of total protein extracts from each sample were denatured in 4X Laemmli Sample buffer (#1610747, Bio-Rad Laboratories) before SDS-PAGE and used for Immunoblot analysis. Primary antibodies used were anti-rabbit Nrf2 (NBP1-32822, Novusbio), anti-rabbit CIITA (orb182642, Biorbyt) and anti-mouse α-tubulin (T5168, Sigma-Aldrich) whereas specific secondary HRP conjugated IgG antibodies were Rabbit (sc2357, Santa Cruz Biotechnology) Mouse (sc516102, Santa Cruz Biotechnology). ClarityMax ECL reagents (#1705062, Bio-Rad laboratories) was used to detect signals with Azure Byosystem (Azure Biosystems). Densitometric analysis for protein quantification was performed with ImageJ blot analyzer software (ImageJ 1.53 k; https://imagej.nih.gov/ij/).

#### Analysis of THP-1 macrophages IL-6 secretion

CM biological effect on human macrophages was performed by assessing the IL-6 release through an ELISA assay. In detail, THP-1-derived macrophages were treated with the CM diluted 1:1, with complete RPMI medium for 24 h. Thereafter, the supernatants were collected, centrifugated at 3000× g for 10 min at 4°C, filtered using a 0.2 μm Ministart sterile filter (#11740966, Sartorius), and stored at −80°C until usage. These cellular supernatants were used to quantify the concentrations of released IL-6 using the Human IL-6 Uncoated ELISA Kit assay (#88-7066-88, Invitrogen) according to the manufacturer’s directions. The plates were read at 450 nm and the sensitivity of the used ELISA assay was in the range 2–200 pg/mL.

#### PBMCs proliferation assay

The immunomodulatory activities of eAECs and mAECs were analyzed on PBMCs by testing their proliferation. 2 × 10ˆ^5^ of PHA stimulated PBMCs were cultured for 72 h with CM derived from immune-activated eAECs amd mAECs (LPS stimulus) following targeting silencing (CIITA for eAECs; NRF2 for mAECs) and CM derived from non-tageting control (NTC for both eAECs and mAECs). PBMCs proliferation was assessed by using CellTiter96 Aqueous One Solution Cell Proliferation Assay following manifacturer’s instruction (G3582, Promega).

### Quantification and statistical analysis

Data were analyzed and plotted using Microsoft Excel (Version 16.71/23031200) and/or GraphPad Prism (Version 9.0.0) softwares. Statistical analysis of data related to Real time qPCR, Immunoblot and Immune assays was performed using Student’s *t* test or one-way ANOVA followed by a Dunnett test. Data generated with NGS were stastistically analyzed with False Discovery Rate (FDR) calculation. Differences were considered significant when p *≤* 0.05. Statistical details of experiments can be found in the figure legends. At least three independent experiments were performed for all of the assays.

## Data Availability

RNA-seq data have been deposited at SRA and are publicly available as of the date of publication. Accession numbers are listed in the key resources table. This paper does not report the original code. Any additional information required to reanalyze the data reported in this paper is available from the lead contact upon request.

## Supplemental information

Document S1. Figures S1 and S2 and Tables S1, S5, S9, and S10

Table S2. DEGs of the study, related to Figure 1 List of over-expressed and under-expressed DEGs of the three pairwise comparisons. First sheet: LPS-eAECs vs. eAECs. Second sheet: LPS-mAECs vs. mAECs. Third sheet: LPS-mAECs vs. LPS-eAECs.

Table S3. GO analysis, related to Figure 1 List of GO terms enriched (biological processes, cellular components, and molecular functions ontologies) for the three pairwise comparisons. First sheet: LPS-eAECs vs. eAECs. Second sheet: LPS-mAECs vs. mAECs. Third sheet: LPS-mAECs vs. LPS-eAECs.

Table S4. List of KEGG pathways enriched for the three pairwise comparisons, related to Figure 1 First sheet: LPS-eAECs vs. eAECs. Second sheet: LPS-mAECs vs. mAECs. Third sheet: LPS-mAECs vs. LPS-eAECs.

Table S6. Network controllers of each experimental group, related to Table 1 List of hub and bottleneck node classification for the three pairwise comparisons. First and second sheets: respectively, bottlenecks and hubs of LPS-eAECs vs. eAECs. Third and fourth sheets: respectively, bottlenecks and hubs of LPS-mAECs vs. mAECs. Fifth and sixth sheets: respectively, bottlenecks and hubs of LPS-mAECs vs. LPS-eAECs.

Table S7. Functional modules identification by ClueGO Cytoscape plug-in, related to Figure 2 ClueGO-based module extraction related to GO and KEGG categories of the three pairwise comparisons. First sheet: LPS-eAECs vs. eAECs. Second sheet: LPS-mAECs vs. mAECs. Third sheet: LPS-mAECs vs. LPS-eAECs.

Table S8. Key driver genes of the study, related to Figure 3 Genes with both hub and bottleneck roles for the three pairwise comparisons were reported. Features such as date hub, DEG, ClueGO Cytoscape module, and related KEGG pathway were also stated. The following abbreviations were adopted: B, bottleneck; H, hub; Dh, date hub; D, DEG; M, module. KEGG pathways of origin were also associated. First sheet: LPS-eAECs vs. eAECs. Second sheet: LPS-mAECs vs. mAECs. Third sheet: LPS-mAECs vs. LPS-eAECs.

Data S1. GO grouped according to semantic similarity parameters, related to Figure 1 REVIGO-based semantic similarity analysis of GO terms (biological processes, cellular components, and molecular functions ontologies) related to the three pairwise comparisons. First sheet: LPS-eAECs vs. eAECs. Second sheet: LPS-mAECs vs. mAECs. Third sheet: LPS-mAECs vs. LPS-eAECs.

## References

[1] Amack J.D. (2021). Cellular dynamics of EMT: lessons from live in vivo imaging of embryonic development. Cell Commun. Signal..

[2] Nieto M.A., Huang R.Y.Y.J., Jackson R.A.A., Thiery J.P.P. (2016). EMT: 2016. Cell.

[3] Kim D.H., Xing T., Yang Z., Dudek R., Lu Q., Chen Y.-H. (2017). Epithelial Mesenchymal Transition in Embryonic Development, Tissue Repair and Cancer: A Comprehensive Overview. J. Clin. Med..

[4] Suarez-Carmona M., Lesage J., Cataldo D., Gilles C. (2017). EMT and inflammation: inseparable actors of cancer progression. Mol. Oncol..

[5] Derynck R., Weinberg R.A. (2019). EMT and Cancer: More Than Meets the Eye. Dev. Cell.

[6] Vitucci D., Imperlini E., Arcone R., Alfieri A., Canciello A., Russomando L., Martone D., Cola A., Labruna G., Orrù S. (2018). Serum from differently exercised subjects induces myogenic differentiation in LHCN-M2 human myoblasts. J. Sports Sci..

[7] Terry S., Savagner P., Ortiz-Cuaran S., Mahjoubi L., Saintigny P., Thiery J.P., Chouaib S. (2017). New insights into the role of EMT in tumor immune escape. Mol. Oncol..

[8] Jiang Y., Zhan H. (2020). Communication between EMT and PD-L1 signaling: New insights into tumor immune evasion. Cancer Lett..

[9] Ma H.Y., Liu X.Z., Liang C.M. (2016). Inflammatory microenvironment contributes to epithelial-mesenchymal transition in gastric cancer. World J. Gastroenterol..

[10] Zhan H.X., Zhou B., Cheng Y.G., Xu J.W., Wang L., Zhang G.Y., Hu S.Y. (2017). Crosstalk between stromal cells and cancer cells in pancreatic cancer: New insights into stromal biology. Cancer Lett..

[11] Lay K., Yuan S., Gur-Cohen S., Miao Y., Han T., Naik S., Pasolli H.A., Larsen S.B., Fuchs E. (2018). Stem cells repurpose proliferation to contain a breach in their niche barrier. Elife.

[12] Harrison O.J., Linehan J.L., Shih H.Y., Bouladoux N., Han S.J., Smelkinson M., Sen S.K., Byrd A.L., Enamorado M., Yao C. (2019). Commensal-specific T cell plasticity promotes rapid tissue adaptation to injury. Science.

[13] Ordovas-Montanes J., Dwyer D.F., Nyquist S.K., Buchheit K.M., Vukovic M., Deb C., Wadsworth M.H., Hughes T.K., Kazer S.W., Yoshimoto E. (2018). Allergic inflammatory memory in human respiratory epithelial progenitor cells. Nature.

[14] Hewitt R.J., Lloyd C.M. (2021). Regulation of immune responses by the airway epithelial cell landscape. Nat. Rev. Immunol..

[15] Barboni B., Russo V., Berardinelli P., Mauro A., Valbonetti L., Sanyal H., Canciello A., Greco L., Muttini A., Gatta V. (2018). Placental Stem Cells from Domestic Animals: Translational Potential and Clinical Relevance. Cell Transplant..

[16] Lindemans C.A., Calafiore M., Mertelsmann A.M., O’Connor M.H., Dudakov J.A., Jenq R.R., Velardi E., Young L.F., Smith O.M., Lawrence G. (2015). Interleukin-22 promotes intestinal-stem-cell-mediated epithelial regeneration. Nature.

[17] Canciello A., Teti G., Mazzotti E., Falconi M., Russo V., Giordano A., Barboni B. (2020). Progesterone Prolongs Viability and Anti-inflammatory Functions of Explanted Preterm Ovine Amniotic Membrane. Front. Bioeng. Biotechnol..

[18] Niknejad H., Paeini-Vayghan G., Tehrani F.A., Khayat-Khoei M., Peirovi H. (2013). Side dependent effects of the human amnion on angiogenesis. Placenta.

[19] Litwiniuk M., Grzela T. (2014). Amniotic membrane: New concepts for an old dressing. Wound Repair Regen..

[20] Canciello A., Greco L., Russo V., Barboni B. (2018). Amniotic Epithelial Cell Culture. Methods Mol. Biol..

[21] Kjaergaard N., Hein M., Hyttel L., Helmig R.B., Schønheyder H.C., Uldbjerg N., Madsen H. (2001). Antibacterial properties of human amnion and chorion in vitro. Eur. J. Obstet. Gynecol. Reprod. Biol..

[22] Ramuta T.Ž., Kreft M.E. (2018). Human Amniotic Membrane and Amniotic Membrane–Derived Cells: How Far Are We from Their Use in Regenerative and Reconstructive Urology?. Cell Transplant..

[23] Canciello A., Russo V., Berardinelli P., Bernabò N., Muttini A., Mattioli M., Barboni B. (2017). Progesterone prevents epithelial-mesenchymal transition of ovine amniotic epithelial cells and enhances their immunomodulatory properties. Sci. Rep..

[24] Mauro A., Sanyal H., Canciello A., Berardinelli P., Russo V., Bernabò N., Valbonetti L., Barboni B. (2019). In Vitro Effect of Estradiol and Progesterone on Ovine Amniotic Epithelial Cells. Stem Cells Int..

[25] Di Lollo V., Canciello A., Orsini M., Bernabò N., Ancora M., Di Federico M., Curini V., Mattioli M., Russo V., Mauro A. (2020). Transcriptomic and computational analysis identified LPA metabolism, KLHL14 and KCNE3 as novel regulators of Epithelial-Mesenchymal Transition. Sci. Rep..

[26] Richardson L.S., Taylor R.N., Menon R. (2020). Reversible EMT and MET mediate amnion remodeling during pregnancy and labor. Sci. Signal..

[27] Bray N.L., Pimentel H., Melsted P., Pachter L. (2016). Near-optimal probabilistic RNA-seq quantification. Nat. Biotechnol..

[28] Russo V., Mauro A., Peserico A., Di Giacinto O., Khatib M.E., Citeroni M.R., Rossi E., Canciello A., Mazzotti E., Barboni B. (2022). Tendon Healing Response Is Dependent on Epithelial–Mesenchymal–Tendon Transition State of Amniotic Epithelial Stem Cells. Biomedicines.

[29] Schumacher A., Costa S.-D., Zenclussen A.C. (2014). Endocrine Factors Modulating Immune Responses in Pregnancy. Front. Immunol..

[30] León Machado J.A., Steimle V. (2021). The mhc class ii transactivator ciita: Not (quite) the odd-one-out anymore among nlr proteins. Int. J. Mol. Sci..

[31] Miki T., Strom S.C. (2006). Amnion-derived pluripotent/multipotent stem cells. Stem Cell Rev..

[32] Barboni B., Curini V., Russo V., Mauro A., Di Giacinto O., Marchisio M., Alfonsi M., Mattioli M. (2012). Indirect Co-Culture with Tendons or Tenocytes Can Program Amniotic Epithelial Cells towards Stepwise Tenogenic Differentiation. PLoS One.

[33] Martin B.K., Chin K.C., Olsen J.C., Skinner C.A., Dey A., Ozato K., Ting J.P. (1997). Induction of MHC class I expression by the MHC class II transactivator CIITA. Immunity.

[34] Gobin S.J., Peijnenburg A., Keijsers V., Van Den Elsen P.J. (1997). Site α is crucial for two routes of IFNγ-induced MHC class I transactivation: the ISRE-mediated route and a novel pathway involving CIITA. Immunity.

[35] Fathi I., Miki T. (2021). Human Amniotic Epithelial Cells Secretome: Components, Bioactivity, and Challenges. Front. Med..

[36] Yang P.J., Guo L.H., Yu L.Y., Yuan W.X., Liu J., Li J.Y., Tan B., Qiu C., Zhu X.L., Qiu C. (2018). Biological characterization of human amniotic epithelial cells in a serum-free system and their safety evaluation. Acta Pharmacol. Sin..

[37] Greco L., Russo V., Rapino C., Germanio C., Di Fezza F., Bernabò N., Berardinelli P., Peserico A., Fazio D., Maccarrone M. (2020). Characterization of Endocannabinoid System and Interleukin Profiles in Ovine AEC: Cannabinoid Receptors Type-1 and Type-2 as Key Effectors of Pro-Inflammatory Response. Cells.

[38] Samarajiwa S.A., Forster S., Auchettl K., Hertzog P.J. (2009). INTERFEROME: The database of interferon regulated genes. Nucleic Acids Res..

[39] Harrison A.R., Moseley G.W. (2020). The Dynamic Interface of Viruses with STATs. J. Virol..

[40] Nan J., Wang Y., Yang J., Stark G.R. (2018). IRF9 and unphosphorylated STAT2 cooperate with NF-κB to drive IL6 expression. Proc. Natl. Acad. Sci. USA.

[41] Parolini O., Souza-Moreira L., O’Valle F., Magatti M., Hernandez-Cortes P., Gonzalez-Rey E., Delgado M. (2014). Therapeutic effect of human amniotic membrane-derived cells on experimental arthritis and other inflammatory disorders. Arthritis Rheumatol..

[42] Pianta S., Bonassi Signoroni P., Muradore I., Rodrigues M.F., Rossi D., Silini A., Parolini O. (2015). Amniotic membrane mesenchymal cells-derived factors skew T cell polarization toward Treg and downregulate Th1 and Th17 cells subsets. Stem Cell Rev. Rep..

[43] Rackaityte E., Halkias J. (2020). Mechanisms of Fetal T Cell Tolerance and Immune Regulation. Front. Immunol..

[44] Canciello A., Cerveró-Varona A., Peserico A., Mauro A., Russo V., Morrione A., Giordano A., Barboni B. (2022). “In medio stat virtus”: Insights into hybrid E/M phenotype attitudes. Front. Cell Dev. Biol..

[45] Chen C.-H. (2020). Xenobiotic Metabolic Enzymes: Bioactivation and Antioxidant Defense.

[46] Gautheron J., Jéru I. (2021). The multifaceted role of epoxide hydrolases in human health and disease. Int. J. Mol. Sci..

[47] Saha S., Buttari B., Panieri E., Profumo E., Saso L. (2020). An Overview of Nrf2 Signaling Pathway and Its Role in Inflammation. Molecules.

[48] Manna S.K., Kuo M.T., Aggarwal B.B. (1999). Overexpression of γ-glutamylcysteine synthetase suppresses tumor necrosis factor-induced apoptosis and activation of nuclear transcription factor-kappa B and activator protein-1. Oncogene.

[49] Soares M.P., Seldon M.P., Gregoire I.P., Vassilevskaia T., Berberat P.O., Yu J., Tsui T.-Y., Bach F.H. (2004). Heme Oxygenase-1 Modulates the Expression of Adhesion Molecules Associated with Endothelial Cell Activation. J. Immunol..

[50] Ganesh Yerra V., Negi G., Sharma S.S., Kumar A. (2013). Potential therapeutic effects of the simultaneous targeting of the Nrf2 and NF-κB pathways in diabetic neuropathy. Redox Biol..

[51] Jing Y.Y., Han Z.P., Sun K., Zhang S.S., Hou J., Liu Y., Li R., Gao L., Zhao X., Zhao Q.D. (2012). Toll-like receptor 4 signaling promotes epithelial-mesenchymal transition in human hepatocellular carcinoma induced by lipopolysaccharide. BMC Med..

[52] Huang T., Chen Z., Fang L. (2013). Curcumin inhibits LPS-induced EMT through downregulation of NF-?B-Snail signaling in breast cancer cells. Oncol. Rep..

[53] Xiao K., He W., Guan W., Hou F., Yan P., Xu J., Zhou T., Liu Y., Xie L. (2020). Mesenchymal stem cells reverse EMT process through blocking the activation of NF-κB and Hedgehog pathways in LPS-induced acute lung injury. Cell Death Dis..

[54] Qin Y., Zhao P., Chen Y., Liu X., Dong H., Zheng W., Li C., Mao X., Li J. (2020). Lipopolysaccharide induces epithelial–mesenchymal transition of alveolar epithelial cells cocultured with macrophages possibly via the JAK2/STAT3 signaling pathway. Hum. Exp. Toxicol..

[55] Fu X.Q., Liu B., Wang Y.P., Li J.K., Zhu P.L., Li T., Tse K.W., Chou J.Y., Yin C.L., Bai J.X. (2020). Activation of STAT3 is a key event in TLR4 signaling-mediated melanoma progression. Cell Death Dis..

[56] Boudhraa Z., Carmona E., Provencher D., Mes-Masson A.M. (2020). Ran GTPase: A Key Player in Tumor Progression and Metastasis. Front. Cell Dev. Biol..

[57] Bros M., Haas K., Moll L., Grabbe S. (2019). RhoA as a Key Regulator of Innate and Adaptive Immunity. Cells.

[58] Svensmark J.H., Brakebusch C. (2019). Rho GTPases in cancer: friend or foe?. Oncogene.

[59] Fucikova J., Kepp O., Kasikova L., Petroni G., Yamazaki T., Liu P., Zhao L., Spisek R., Kroemer G., Galluzzi L. (2020). Detection of immunogenic cell death and its relevance for cancer therapy. Cell Death Dis..

[60] Bond S., Lopez-Lloreda C., Gannon P.J., Akay-Espinoza C., Jordan-Sciutto K.L. (2020). The integrated stress response and phosphorylated eukaryotic initiation factor 2α in neurodegeneration. J. Neuropathol. Exp. Neurol..

[61] Liang Q., Liu Z., Zhu C., Wang B., Liu X., Yang Y., Lv X., Mu H., Wang K. (2018). Intrahepatic T-Cell Receptor β Immune Repertoire Is Essential for Liver Regeneration. Hepatology.

[62] Werner L., Nunberg M.Y., Rechavi E., Lev A., Braun T., Haberman Y., Lahad A., Shteyer E., Schvimer M., Somech R. (2019). Altered T cell receptor beta repertoire patterns in pediatric ulcerative colitis. Clin. Exp. Immunol..

[63] Steidl C., Shah S.P., Woolcock B.W., Rui L., Kawahara M., Farinha P., Johnson N.A., Zhao Y., Telenius A., Neriah S.B. (2011). MHC class II transactivator CIITA is a recurrent gene fusion partner in lymphoid cancers. Nature.

[64] Huang X.P., Ludke A., Dhingra S., Guo J., Sun Z., Zhang L., Weisel R.D., Li R.K. (2016). Class II transactivator knockdown limits major histocompatibility complex II expression, diminishes immune rejection, and improves survival of allogeneic bone marrow stem cells in the infarcted heart. FASEB J..

[65] Yao X., Huang J., Zhong H., Shen N., Faggioni R., Fung M., Yao Y. (2014). Targeting interleukin-6 in inflammatory autoimmune diseases and cancers. Pharmacol. Ther..

[66] Jordan S.C., Choi J., Kim I., Wu G., Toyoda M., Shin B., Vo A. (2017). Interleukin-6, A cytokine critical to mediation of inflammation, autoimmunity and allograft rejection: Therapeutic implications of IL-6 receptor blockade. Transplantation.

[67] Villarino A.V., Kanno Y., O’Shea J.J. (2017). Mechanisms and consequences of Jak-STAT signaling in the immune system. Nat. Immunol..

[68] Mogensen T.H. (2018). IRF and STAT transcription factors - From basic biology to roles in infection, protective immunity, and primary immunodeficiencies. Front. Immunol..

[69] Liang Y., Jing Z., Deng H., Li Z., Zhuang Z., Wang S., Wang Y. (2015). Soluble epoxide hydrolase inhibition ameliorates proteinuria-induced epithelial-mesenchymal transition by regulating the PI3K-Akt-GSK-3β signaling pathway. Biochem. Biophys. Res. Commun..

[70] Václavíková R., Hughes D.J., Souček P. (2015). Microsomal epoxide hydrolase 1 (EPHX1): Gene, structure, function, and role in human disease. Gene.

[71] Bocci F., Tripathi S.C., Vilchez Mercedes S.A., George J.T., Casabar J.P., Wong P.K., Hanash S.M., Levine H., Onuchic J.N., Jolly M.K. (2019). NRF2 activates a partial epithelial-mesenchymal transition and is maximally present in a hybrid epithelial/mesenchymal phenotype. Integr. Biol..

[72] He F., Ru X., Wen T. (2020). NRF2, a transcription factor for stress response and beyond. Int. J. Mol. Sci..

[73] Yang Y., Huycke M.M., Herman T.S., Wang X. (2016). Glutathione S-transferase alpha 4 induction by activator protein 1 in colorectal cancer. Oncogene.

[74] Park H.J., Kim M.J., Rothenberger C., Kumar A., Sampson E.M., Ding D., Han C., White K., Boyd K., Manohar S. (2019). GSTA4 mediates reduction of cisplatin ototoxicity in female mice. Nat. Commun..

[75] Louie S.M., Grossman E.A., Crawford L.A., Ding L., Camarda R., Huffman T.R., Miyamoto D.K., Goga A., Weerapana E., Nomura D.K. (2016). GSTP1 Is a Driver of Triple-Negative Breast Cancer Cell Metabolism and Pathogenicity. Cell Chem. Biol..

[76] Cui J., Li G., Yin J., Li L., Tan Y., Wei H., Liu B., Deng L., Tang J., Chen Y., Yi L. (2020). GSTP1 and cancer: Expression, methylation, polymorphisms and signaling (Review). Int. J. Oncol..

[77] Bi X., Li J., Fan X., Zhou J., Jiang B., Yang Z., Luo L., Yin Z. (2020). GSTP1 Inhibits LPS-Induced Inflammatory Response Through Regulating Autophagy in THP-1 Cells. Inflammation.

[78] Dufrusine B., Di Francesco A., Oddi S., Scipioni L., Angelucci C.B., D’Addario C., Serafini M., Häfner A.K., Steinhilber D., Maccarrone M., Dainese E. (2019). Iron-Dependent Trafficking of 5-Lipoxygenase and Impact on Human Macrophage Activation. Front. Immunol..

[79] Conesa A., Madrigal P., Tarazona S., Gomez-Cabrero D., Cervera A., McPherson A., Szcześniak M.W., Gaffney D.J., Elo L.L., Zhang X., Mortazavi A. (2016). A survey of best practices for RNA-seq data analysis. Genome Biol..

[80] Bolger A.M., Lohse M., Usadel B. (2014). Trimmomatic: A flexible trimmer for Illumina sequence data. Bioinformatics.

[81] Hunt S.E., McLaren W., Gil L., Thormann A., Schuilenburg H., Sheppard D., Parton A., Armean I.M., Trevanion S.J., Flicek P. (2018). Ensembl Variation Resources. Database (Oxford).

[82] McCarthy D.J., Chen Y., Smyth G.K. (2012). Differential expression analysis of multifactor RNA-Seq experiments with respect to biological variation. Nucleic Acids Res..

[83] Smedley D., Haider S., Durinck S., Pandini L., Provero P., Allen J., Arnaiz O., Awedh M.H., Baldock R., Barbiera G. (2015). The BioMart community portal: An innovative alternative to large, centralized data repositories. Nucleic Acids Res..

[84] Supek F., Bošnjak M., Škunca N., Šmuc T. (2011). Revigo summarizes and visualizes long lists of gene ontology terms. PLoS One.

[85] Yu G., Wang L.G., Han Y., He Q.Y. (2012). ClusterProfiler: An R package for comparing biological themes among gene clusters. Omi. A J. Integr. Biol..

[86] Robinson M.D., McCarthy D.J., Smyth G.K. (2010). edgeR: A Bioconductor package for differential expression analysis of digital gene expression data. Bioinformatics.

[87] Zhang J.D., Wiemann S. (2009). KEGGgraph: A graph approach to KEGG PATHWAY in R and bioconductor. Bioinformatics.

[88] Shannon P., Markiel A., Ozier O., Baliga N.S., Wang J.T., Ramage D., Amin N., Schwikowski B., Ideker T. (2003). Cytoscape: A software Environment for integrated models of biomolecular interaction networks. Genome Res..

[89] Bernabò N., Mattioli M., Barboni B. (2015). Signal transduction in the activation of spermatozoa compared to other signalling pathways: A biological networks study. Int. J. Data Min. Bioinform..

[90] Bernabò N., Greco L., Ordinelli A., Mattioli M., Barboni B. (2015). Capacitation-Related Lipid Remodeling of Mammalian Spermatozoa Membrane Determines the Final Fate of Male Gametes: A Computational Biology Study. Omi. A J. Integr. Biol..

[91] Chin C.H., Chen S.H., Wu H.H., Ho C.W., Ko M.T., Lin C.Y. (2014). cytoHubba: Identifying hub objects and sub-networks from complex interactome. BMC Syst. Biol..

[92] Bader G.D., Hogue C.W.V. (2003). An automated method for finding molecular complexes in large protein interaction networks. BMC Bioinf..

