# Supplementary material for: Unveiling the immunomodulatory shift: Epithelial-mesenchymal transition Alters immune mechanisms of amniotic epithelial cells
Source: iScience. 2023 Aug 9;26(9):107582. doi: 10.1016/j.isci.2023.107582 (PMC10481295; doi:10.1016/j.isci.2023.107582)
Supplement: Document S1. Figures S1 and S2 and Tables S1, S5, S9, and S10 [file mmc1.pdf]

iScience, Volume *26*

Supplemental information

Unveiling the immunomodulatory shift: Epithelial-mesenchymal transition Alters immune mechanisms of amniotic epithelial cells

Valeria Di Lollo, Angelo Canciello, Alessia Peserico, Massimiliano Orsini, Valentina Russo, Adrián Cerveró-Varona, Beatrice Dufrusine, Mohammad El Khatib, Valentina Curini, Annunziata Mauro, Paolo Berardinelli, Cathy Tournier, Massimo Ancora, Cesare Cammà, Enrico Dainese, Luana Fiorella Mincarelli, and Barbara Barboni

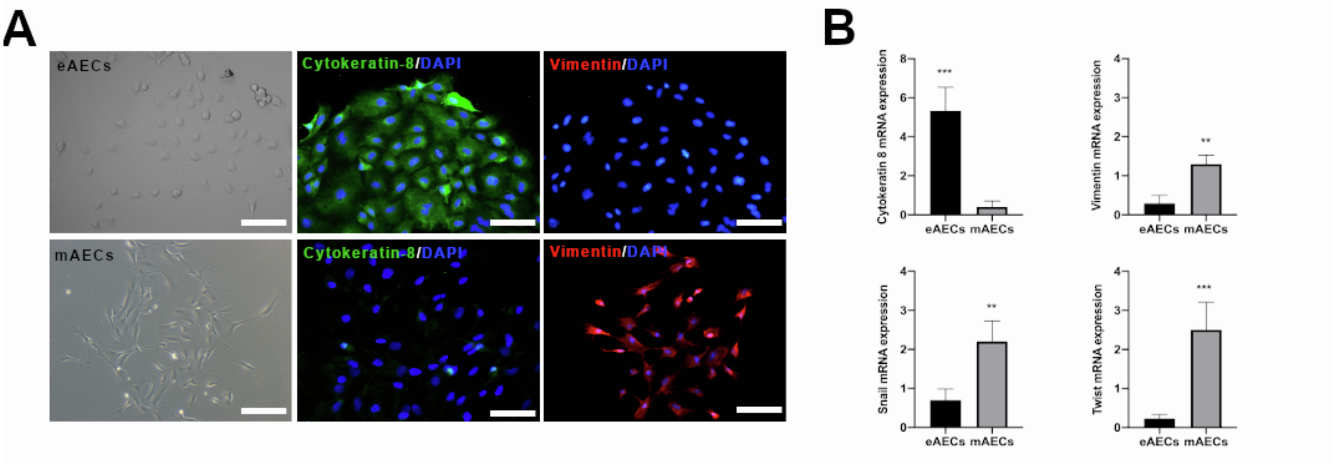

Figure S1. eAECs and mAECs phenotype characterization, related to Figure 1. (A) Phenotypic characterization of eAECs and mAECs by phase constast microscopy and immunofluorescence analysis of Cytokeratin- 8 and Vimentin. Scale Bar: 50 µm. **(B)** Real-time qPCR Cytokeratin-8, Vimentin, Snail and Twist mRNA expression in mAECs and eAECs. Results from gene expression analyses are the mean ± SD, of the 2^-ΔΔCt values from n = 3 independent experiments. Significant changes vs. were represented with * = $p < 0.05$; ** = $p < 0.01$; *** = $p < 0.001$; **** = $p < 0.0001$. Student's t-tail test was used.

A

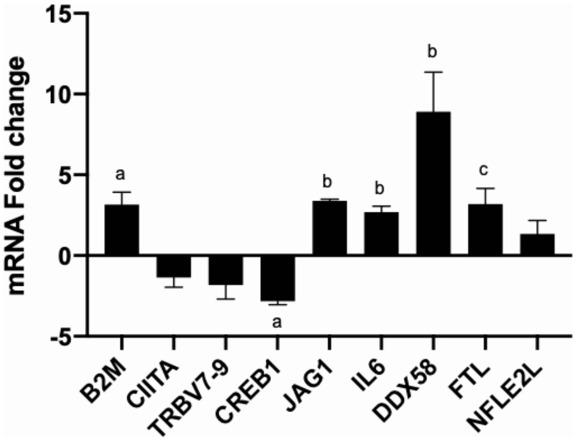

B

Gene Symbol	Gene Description	Topological features			Expression features	
		Hub	Bottleneck	Local hub	DEG	Significant
**B2M**	beta 2 microglobulina				x	x
**CIITA**	Class II Major Histocompatibility Complex Transactivator	x	x	x		
**TRB**	T-cell receptor beta chain	x	x	x		
**CREB1**	CAMP responsive element binding protein	x	x		x	
**JAG1**	Jagged Canonical Notch Ligand 1				x	
**IL6**	Interleukin 6	x	x		x	x
**DDX58**	DExD/H-Box Helicase 58		x		x	x
**FTL**	Ferritin light chain				x	x
**NFE2L2**	Nuclear Factor, Erythroid 2 Like 2	x		x		

Figure S2. Real time qPCR validation of NGS data, related to Table 2. (A) Real-Time qPCR validation of most representative genes. Fold-changes are related to the mean ± SD of 2^- ∆∆Ct values obtained in n = 3 independent experiments. The significance of B2M, CIITA, TRBV7-9 and CREB data related to LPS-treated eAECs ($p < 0.05$) was statistically analyzed vs. eAECs and indicated with (a). The significance of JAG1, IL6 and DDX58 data related to LPS-treated mAECs ($p < 0.05$) was statistically analyzed vs. mAECs and indicated with (b). The significance of FTL and NFLE2L data related to LPS-treated mAECs ($p < 0.05$) was statistically analyzed vs. LPS-treated eAECs and indicated with (c) **(B)** the list of genes selected for the Real-Time qPCR validation and their topological and/or expression features.

	eAECs			LPS - eAECs			mAECs			LPS - mAECs		
**Comparisons**	**1**	**2**	**3**	**1**	**2**	**3**	**1**	**2**	**3**	**1**	**2**	**3**
**Raw reads (Million)**	30,5	29,2	35,3	31,3	30,2	35,0	32,4	30,4	29,8	30,8	28,2	32,8
**Clean reads (Million)**	30,4	29,1	35,2	31,2	30,0	34,9	32,3	30,3	29,8	30,8	28,2	32,8
**Bases before filtering (Gb)**	2,3	2,2	2,6	2,3	2,2	2,6	2,4	2,3	2,2	2,3	2,1	2,4
**Bases after filtering (Gb)**	1,9	1,8	2,2	2,0	1,9	2,2	2,0	1,9	1,9	1,9	1,8	2,1
**Q30 before filtering (%)**	97%	96.9%	97%	97.1%	97.0 %	97.1 %	97.1 %	97.1 %	97.1 %	97.1 %	96.9 %	97.0 %
**Q30 after filtering (%)**	97.1 %	96.9%	97.1%	97.1%	97.1%	97.1%	97.2%	97.1%	97.1%	97.1%	96.9%	97.0%
**Mean Len before filtering (bp)**	75	75	75	75	75	75	75	75	75	75	75	75
**Mean Length After Filtering (bp)**	64	64	64	64	64	64	64	64	64	64	64	64
**Processed**	30,4	35,3	29,1	31,2	30,1	34,9	32,3	30,3	29,8	30,8	28,2	32,8
**Pseudoaligned**	13,8 (45.5%)	17,3 (49.1%)	13,4 (46%)	14,2 (45.5%)	13,5 (44.8 %)	16,6 (47.5 %)	15,1 (46.6 %)	13,2 (43.7 %)	13,6 (45.8 %)	13,1 (42.5 %)	11,5 (40.7 %)	14,7 (44.9 %)
**Unique**	12,1 (39.8 %)	14,4 (41.0%)	11,4 (39.2%)	12,6 (40.2%)	11,4 (38.0%)	13,9 (39.6%)	12,9 (39.9%)	11,4 (37.5 %)	11,6 (38.8 %)	11,3 (36.8 %	10,0 (35.5 %)	12,5 (38.2 %)

Table S1. Table of RNA sequencing and mapping statistics, related to Figure 1. The table summarizes the performance of the RNA sequencing and mapping for the three biological replicates in each cell population (eAECs and mAECs phenotypes).

Shared KEGG Pathways	LPS-eAECs *vs* eAECs	LPS-mAECs *vs* mAECs	LPS-mAECs vs LPS-eAECs	Total number of DEGs	Shared DEG number	Shared DEG %
**Influenza A**	X	X	X	23	1	4
**Epstein-Barr virus infection**	X	X		22	2	9
**C-type leptin receptor**	X	X		12	2	17
**Interleukin 17 signaling**	X	X		10	1	10
**Apelin signaling**	X	X		12	1	8
**Pathways in cancer**		X	X	29	3	10
**Spliceosome**		X	X	15	0	0
**Measles**		X	X	13	2	15
**Ferroptosis**		X	X	6	1	17
**Mineral absorbtion**		X	X	8	2	25
**Ribosome**	X		X	22	0	0
**RNA transport**	X		X	18	1	6
**Herpex Simplex virus 1 infection**	X		X	15	0	0

Table S5. KEGG pathways sharing in all groups, related to Figure 1. The table shows KEGG pathways shared across all the experimental pairwise comparisons and the number of DEGs subtending each pathway. Common DEGs were reported as the number and percentage (%) of shared DEGs.

Gene Name	Gene Name	Target Sequence (5’-3’)
**NRF2 Smart pool**<br>Cat# FE52L001000005	**NRF2_1**	TGACAGAAATTGACAGTTA
	**NRF2_2**	AGAGATGAGCTACGGGCAA
	**NRF2_3**	CTGCTGATTTAGACGATAT
	**NRF2_4**	CCAAGGAGCAATTCAACGA
**CIITA Smart pool**<br>Cat# FE51L0010000050	**CIITA_1**	AGAAGGTGCTCACGAGGTA
	**CIITA_2**	GGAGAAGAGCAGCGGGAAG
	**CIITA_3**	CCAAGTGGCCTGAGAGCGT
	**CIITA_4**	AGGCTGAGACCTACGTGAA
**ON TARGETplus Non-targeting Pool**<br>Cat# FE5D0018101005	**NTC_1**	UGGUUUACAUGUCGACUAA
	**NTC_2**	UGGUUUACAUGUUUGUGUGA
	**NTC_3**	UGGUUUACAUGUUUUCUGA
	**NTC_4**	UGGUUUACAUGUUUUCCUA

Table S9. Target sequence of siRNAs, related to STAR Methods. Target sequences of siRNA against CIITA, Nrf2 and target sequences of siRNA NTC. Both Nr2 and CIITA genes have been targeted by means of four gene-specific siRNAs, herein referred as “pool” following manufacturer’s instruction.

Gene	Forward sequence	Reverse sequence	Annealing Temperature (°C)
**B2M**	5′-CTGTCGCTGTCTGGACTGG-3′	5′-TTTGGCTTTCCATCTTCTGG-3′	61.4
**CIITA**	5′-CAAAGCATGACCGCTGGAAATT-3′	5′-AAACAAACAGGAAATGGAGGCAAA-3′	57
**TRBV7-9**	5′-AGTGTCTCTATGCCTTGCTC-3′	5′-AGTCCTGTCTTCACCCATC-3′	61.7
**CREB1**	5′-CTGGGGTTGTTATGGCGTCT-3′	5′-AGCACTGCCACTCTGTTCTC-3′	60
**JAG1**	5′-TGTTCTCCAAATAACTGTTCCC-3′	5′-AGTTTTGACCCATCCAGCC-3′	61.5
**IL6**	5′-ACCTGGACTTCCTCCAGAAC-3′	5′-TTGAGGACTGCATCTTCTCC-3′	62
**DDx58**	5′-TGGAGAGATAGCTGACAGTG-3′	5′-AGTAAAGATGGAGAGTGATGGG-3′	61.5
**FTL**	5′-CCTTTTTTCCAGTCGCAACC-3′	5′-GATCGCGGACAGCTGAGTG-3′	62.9
**NFE2L2**	5′-CCAGTCTTCAATGCTCCTTC-3′	5′-GTTCCTCCCAAACTTGCTC-3′	61.3
**GAPDH**	5′-TCGGAGTGAACGGATTTGGC-3′	5′-CCGTTCTCTGCCTTGACTGT-3′	64.4
**YWHAZ**	5′-AGACGGAAGGTGCTGAGAAA-3′	5′-CGTTGGGGATCAAGAACTTT-3′	61.3
**Vimentin**	5′-GACCAGCTCACCAACGACA-3′	5′-CTCCTCCTGCAACTTCTCCC-3′	61.2
**Twist**	5′-GCCGGAGACCTAGATGTCATTG-3′	5′-GCCGGAGACCTAGATGTCATTG-3′	62
**Snail**	5′-GTCGTGGGTGGAGAGCTTTG-3′	5′-TGCTGGAAAGTGAGCTCTGG-3′	61.6
**Cytokeratin-8**	5′-CTCAAAGGCCAGGGCTTC-3′	5′-CTTGGCCTGGCATCCTTGA-3′	61.3

Table S10. Primers for qPCR analysis, related to STAR methods. Sequences of primers and conditions used in real-time qPCR.
